# Comparative Study of Predicting Radical C—H Functionalization Sites in Nitrogen Heteroarenes Using a Radical General‐Purpose Reactivity Indicator and the Radical Fukui Function

**DOI:** 10.1002/jcc.70130

**Published:** 2025-05-19

**Authors:** Yoshio Barrera, James S. M. Anderson

**Affiliations:** ^1^ Instituto de Química Universidad Nacional Autónoma de México México City México

**Keywords:** chemical reactivity, conceptual DFT, Fukui function, general‐purpose reactivity indicator, radical addition reactions, radical chemistry, radical C—H functionalization

## Abstract

The Radical General‐Purpose Reactivity Indicator (R‐GPRI) is a valuable new tool for discerning the most reactive atoms within a molecule undergoing radical attack. In this study, we apply the condensed R‐GPRI and the condensed Radical Fukui Function (RFF) to identify the two most reactive atoms in 14 nitrogen heteroarenes subjected to radical attack by •CF_3_ (trifluoromethyl radical) and •*i*‐Pr (isopropyl radical). The results were compared with available experimental data and calculated activation barriers to comprehensively evaluate the reactivity of these molecules, especially in reactions without isolated products. The outcomes indicate that R‐GPRI is a robust alternative for identifying the most reactive C—H sites in disubstituted nitrogen heteroarenes, outperforming the RFF. We also found that for nitrogen heteroarenes, summing the charges of the hydrogen atoms into the heavier atoms to which they are bonded as computed with the Hirshfeld population scheme enhances the performance of the RFF compared to previous findings. As such, the R‐GPRI appropriately incorporates both charge and RFF contributions. It is also observed that the atoms within molecules that have a small condensed RFF tend to be unreactive in these radical attack reactions. This is even observed in atoms with the largest charge in magnitude (with the appropriate sign) but a small condensed RFF.

## Graphical abstract

This study investigates the precise identification of reactive C—H positions within nitrogen heteroarenes, crucial for synthesizing pharmaceutical compounds. We employed the Radical General‐Purpose Reactivity Indicator (R‐GPRI) and the Radical Fukui Function (RFF) to determine the regioselectivity of nitrogen heteroarenes susceptible to •CF_3_ and •*i*‐Pr attacks. To underscore the efficacy of both models, we compared our results with available experimental and calculated data. Our study highlights the superiority of the R‐GPRI over the RFF in predicting the second major product of these reactions. However, both models have similar performance in elucidating the first most reactive atom.

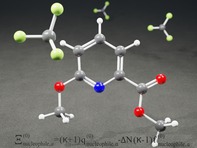

## 1 Introduction

**SCHEME 1:**
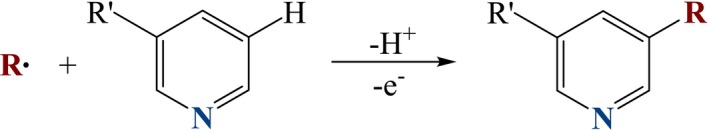
Schematic representation of the radical C—H functionalization on nitrogen heteroarenes.

Radical C—H functionalization on nitrogen heteroarenes involves the substitution of an aromatic C—H bond with a C—R group through a radical reaction [1, 2], in accordance with Scheme 1. The nature of the functionalized site is dictated by the attacking radical.

These reactions have proven to be a convenient methodology for modifying aromatic carbons on heterocycles, leading to the discovery of interesting organic molecules [3, 4] with substantial potential for pharmaceutical applications [5, 6, 7, 8, 9]. In 2013, Baran et al. [10] reported the experimental results of reactions between various nitrogen heteroarenes and radicals. Their observations led them to postulate empirical rules to predict and modulate the regioselectivity of these reactions. Since these principles are based on the intrinsic electronic properties of the nitrogen heteroarenes, Xiong et al. [11] proposed using the condensed Radical Fukui function (RFF) of the atoms, computed using the Hirshfeld population scheme [12] at the B3LYP/6‐31+G** level of theory with the DMSO continuum model, in the heteroarenes as a straightforward alternative method to elucidate the regioselectivity of these molecules. However, the condensed RFF values did not accurately reflect the experimental regioselectivity. In contrast, the condensed nucleophilic attack Fukui function successfully predicted the correct single isopropylation functionalization sites on a set of heteroarenes [11].

Inspired by these observations, our main objective is to address the question: “Can the R‐GPRI predict the correct regioselectivity of nitrogen heteroarenes undergoing radical attack when the RFF fails?” To answer this, we applied the condensed Radical General‐Purpose Reactivity Indicator (R‐GPRI) [13, 14], which can be viewed as an enhanced version of the condensed RFF that includes atomic charges, to identify the first and the second most susceptible aromatic carbon atoms to be functionalized in fourteen nitrogen heteroarenes by •CF_3_ and •*i*‐Pr. The outcomes of the condensed R‐GPRI and condensed RFF were compared with the experimental data reported by Baran et al. [10] and the calculated activation barriers, ${\Delta H}_{298\;K}^{‡}$.

## 2 Theoretical Background

The condensed R‐GPRI (Equations 1 and 2) are modified versions of the original condensed GPRI [15, 16, 17, 18, 19, 20, 21], designed to describe the reactivity of molecules susceptible to radical addition reactions [13, 14]. Developed by Anderson et al. [15, 16, 20], the GPRI models are based on minimizing the interaction energy as reactants approach each other assuming that the transition state (TS) closely resembles the reactants. Thus, the lower the interaction energy at a specific atom, the greater its reactivity. The GPRI models are derived by expanding the change of the electronic energy within the canonical ensemble as a Taylor series in terms of electron number (N) and external potential ($\nu \left(r\right)$) [22, 23, 24]. These models are considered next‐generation reactivity descriptors due to their ability to extend frontier molecular orbital theory [25] by incorporating the Fukui function and electrostatic potential or atomic charges in their condensed forms [26]. The GPRI models are limited by the omission of higher‐order terms in the energy expansion [27], simplifying the attacking molecule as a point‐like charge with a Fukui function, and assuming an early TS [15]. Despite these assumptions the GPRI models have shown excellent performance in predicting reactive sites on molecules undergoing nucleophilic, electrophilic, or radical attack [13, 14, 16, 17, 18, 20, 21, 28, 29, 30].

The condensed R‐GPRI depends on two parameters, κ and ∆N. The first one, κ, is associated with the atomic charge and the Fukui function of the attacking molecule, and ∆N represents the extent of electron transfer, see Appendix B. Therefore, the α atom with the smallest value of Equation (1) is predicted to be the most reactive site in a nucleophile‐like molecule undergoing an electrophilic radical attack at specific values of κ and ∆N,

(1) $$\;\Xi_{\text{nucleophile},\alpha }^{0}=\left(\kappa +1\right)q_{\text{nucleophile},\alpha }^{\left(0\right)}-∆N\left(\kappa -1\right)f_{\text{nucleophile},\alpha }^{\left(0\right)}$$

likewise, the α atom with the smallest value of Equation (2) is predicted to be the most susceptible site in an electrophile‐like molecule undergoing a nucleophilic radical attack at specific values of κ and ∆N,

(2) $$\Xi_{\text{electrophile},\alpha }^{0}=-\left(\kappa +1\right)q_{\text{electrophile},\alpha }^{\left(0\right)}+∆N\left(\kappa -1\right)f_{\text{electrophile},\alpha }^{\left(0\right)}$$

In Equations (1) and (2), $q_{\text{nucleophile},\alpha }^{\left(0\right)}$ and $q_{\text{electrophile},\alpha }^{\left(0\right)}$ represent the atomic charges of each α atom in the ground state (typically neutral) of the target nucleophile and electrophile, respectively. Similarly, $f_{\text{nucleophile},\alpha }^{\left(0\right)}$ and $f_{\text{electrophile},\alpha }^{\left(0\right)}$ denote the condensed RFF, computed using the finite difference approach [31], for each α atom in the nucleophile‐ or electrophile‐like molecule under study, see Equation (5). To assign properties to atoms the condensed charges, condensed Fukui functions and the condensed R‐GPRI are used. These are computed using population schemes [14]. Since the Fukui function has singularities when evaluated at integer electron numbers [32, 33], the condensed Fukui function is typically evaluated from above, below or the average of those [32, 34, 35]. This results in three different interpretations. The condensed Fukui function evaluated from below, $f_{\alpha }^{\left(-\right)}$, Equation (3), is used to identify the most susceptible sites in a nucleophile undergoing an electrophilic attack. Conversely, the Fukui function evaluated from above, $f_{\alpha }^{\left(+\right)}$, Equation (4), is used to determine the reactivity of electrophiles undergoing a nucleophilic attack. The average of both, $f_{\alpha }^{\left(0\right)}$, Equation (5), is used to elucidate the regioselectivity of molecules susceptible to radical attack [35].

(3) $$f_{\alpha }^{\left(-\right)}=q_{\alpha }\left(\rho_{N-1}\right)-q_{\alpha }\left(\rho_{N}\right)$$

(4) $$f_{\alpha }^{\left(+\right)}=q_{\alpha }\left(\rho_{N}\right)-q_{\alpha }\left(\rho_{N+1}\right)$$

(5) $$f_{\alpha }^{\left(0\right)}=\frac{1}{2}\left(f_{\alpha }^{+}+f_{\alpha }^{-}\right)$$

The condensed Fukui functions in Equations ((3), (4), (5)) use the atomic charges, $q_{\alpha }\left(\rho_{N}\right)$, $q_{\alpha }\left(\rho_{N-1}\right)$, and $q_{\alpha }\left(\rho_{N+1}\right)$, which represents the atomic charges of each α atom in the molecule with N electrons (usually a neutral molecule), *N* − 1 electrons (usually a cation), and *N* + 1 electrons (usually an anion), respectively. In this approach, atoms with the highest values from Equations ((3), (4), (5)) are considered as the most susceptible sites to electrophilic, nucleophilic, and radical attack, respectively.

The electron transfer, Δ*N*, in the R‐GPRI is typically considered within the ranges −1≤ΔN≤0 for nucleophile‐like molecules undergoing an electrophilic radical attack, and 0≤ΔN≤1 for electrophile‐like molecules undergoing a nucleophilic radical attack. As detailed by Anderson et al. [15], κ determines the reaction mechanism of the reactions, that is, charge‐controlled, charge‐transfer‐controlled, and in‐between. Generally, κ ranges from −1 to 1, modulating the weight of the two terms in Equations (1) and (2) [14, 15, 16, 17, 18, 21]. Thus, when κ≈−1, the RFF is the dominant term, and the R‐GPRI is predicting the outcome of an electron transfer controlled reaction, when κ≈1, the atomic charge is the dominant term, and R‐GPRI is predicting the outcome of an electrostatic controlled reaction, and in‐between for predicting the outcome of a reaction when both effects are important, κ≈0. Values outside this range predict strongly electrostatically controlled behavior (κ>1) or strongly electron transfer controlled behavior (κ<−1). The condensed R‐GPRI values for each atom α are determined and compared at specific values of Δ*N* and κ, where the atom with the smallest value at a given set of Δ*N* and κ is identified as the most reactive (“first choice”), while the atom with the second smallest values is determined as the second most reactive (“second choice”) under those conditions (given set of Δ*N* and κ). These results are then organized into reactivity transition tables (RTTs) alongside their corresponding atomic labels, see Appendices A and B [16]. Refer to Tables S2–S29 for details.

The radical addition reactions studied here involve neutral nitrogen heteroarenes and radicals (•CF_3_ and •*i*‐Pr), indicating significant electron transfer control behavior [36, 37]. Two areas have been defined in the RTTs for each molecule (Tables S2–S29 in the SM). The first area models the reaction as electron transfer controlled, displayed in a black box in the RTTs, −0.8≤ΔN≤0.2 and −0.4≤κ≤−1, for electrophilic radical attack, Equation (1), and −0.2≤ΔN≤0.8 and −0.4≤κ≤−1, for nucleophilic radical attack, Equation (2). These bounds were efficacious for determining the most reactive atom in our previous work [13, 14]. The second area, displayed in a pink box in the RTTs, models the reactions with stronger electron transfer control than the black box, which is defined by, −0.8≤ΔN≤0.2 and −1.2≤κ≤−1.4 for electrophilic radical attack, Equation (1), and −0.2≤ΔN≤0.8 and −1.2≤κ≤−1.4 for nucleophilic radical attack, Equation (2). Additionally, we consider the extended radical reactivity condition (ERRC) which combines both areas, black box + pink box. The atoms in the majority of these boxes correspond to the expected most reactive/susceptible in the molecule of interest under the modeled conditions as described by the Δ*N* and *κ* values.

Since a radical in organic chemistry is defined as a molecule with an unpaired electron in its valence orbital [38], singly occupied molecular orbital (SOMO) [39], these chemical species can donate electrons (nucleophilic radical) or accept electrons (electrophilic radical) [40] depending on the functional groups attached to the radical center [41]. Electron‐donating groups like alkyl groups increase the nucleophilic character of the radical [42], and electron‐withdrawing groups such as nitrile groups increase the electrophilic character of the radical [43]. Hence, it is crucial to know the overall chemical behavior of the radical with respect to the molecule of interest to elucidate which of the two condensed R‐GPRI equations is the proper one for each reaction. In the literature there are some experimental alternatives to understand the chemical behavior of radicals [40, 44], as well as theoretical methodologies [45, 46, 47]. However, in this study we compared the Mulliken electronegativity (χ) values of each reactant (nitrogen heteroarenes and radicals) [48] to elucidate the overall behavior of the target molecule in relation to the attacking radicals. Within the context of CDFT, χ measures the tendency of a molecule to gain electrons [49],

(6) $$\chi =\frac{I+A}{2}$$

where the χ values are determined as the average of the first vertical ionization energy, I, and the first electron affinity, A [48],

(7) $$I=E\left(N-1\right)-E\left(N\right)$$

(8) $$A=E\left(N\right)-E\left(N+1\right)$$

where $E\left(N\right)$, $E\left(N+1\right)$, and $E\left(N-1\right)$ are the ground state energy of the system with N electrons (usually neutral), with an extra electron (usually an anion), and with an electron removed (usually a cation), respectively. Consequently, comparing the electronegativity values calculated using Equation (6), for the reactants (the radicals and the molecules of interest) provides two situations:

1. If the electronegativity of the radical, $\chi_{\text{radical}}$, is greater than the values of the molecule of interest, $\chi_{\text{molecule}}$, $\chi_{\text{molecule}}<\chi_{\text{radical}}$, the radical tends to gain electrons, thus the radical behaves as an electrophilic radical, Equation (1).
2. If the electronegativity of the radical is less than that of the corresponding molecule, $\chi_{\text{molecule}}>\chi_{\text{radical}}$, the radical tends to donate electrons, thus the radical behaves as a nucleophilic radical, Equation (2).

## 3 Methodology

All computations were conducted using Gaussian 16 Rev. C.01 [50] utilizing density‐functional theory (DFT) [51, 52, 53, 54, 55], employing the B3LYP exchange‐correlation energy functional [56, 57, 58, 59, 60] and the 6‐311++G** basis set [61, 62, 63, 64, 65] within the Kohn‐Sham framework. To calculate the electronegativity and the condensed RFF values of each molecule including the radicals, all the geometries of the neutral (*N* electron) structures were optimized, then single point energy calculations on the optimized structures were performed to obtain the energy and the atomic charges of the (N+1) and (N−1) electron systems.

**SCHEME 2:**
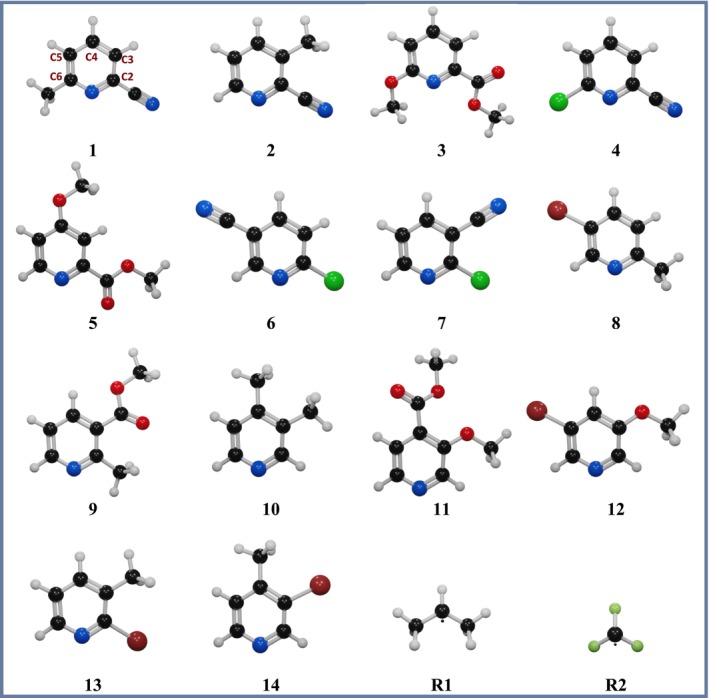
Molecular structure of nitrogen heteroarenes (**1**–**14**) and radicals •*i*‐Pr (**R1**) and •CF_3_ (**R2**). The numbering scheme of the carbon atoms, shown in **1**, is applied to all nitrogen heteroarenes, where C2, C3, and so forth, denote specific carbon positions. Atom colors: black (carbon), red (oxygen), light gray (hydrogen), blue (nitrogen), light green (fluorine), green (chlorine), and burgundy (bromine).

The sensitivity of the condensed RFF and the condensed R‐GPRI was investigated by the current authors [14] using a variety of population schemes at the B3LYP/6‐311++G** as implemented in Gaussian 16 [50]. The Hirshfeld population scheme [12, 14] was found to be the most reliable method for discerning atoms in a molecule that are most susceptible to radical attack within the condensed R‐GPRI and RFF frameworks. Hence, the charges used in this article were computed using the Hirshfeld population scheme at the B3LYP/6–311++G** as implemented in Gaussian 16 [50]. Only charges of the susceptible carbons in the ring of the heteroarenes to radical attack, which correspond to the susceptible atoms to radical C—H functionalization, labeled as C2–C6 [10], were considered; see Scheme 2. Additionally, the contributions of the bonded hydrogens were summed into the corresponding carbon.

In Section 4, we compared the R‐GPRI outcomes with the activation enthalpies, ${\Delta H}_{298\;K}^{‡}$. The TSs for the radical addition reaction on each likely reactive site of the heteroarene and the corresponding radical was computed. Reports have highlighted the sensitivity of calculated activation barriers relative to the chosen level of theory [66, 67, 68, 69]. Waroquier et al. [66] demonstrated that deviations in the activation barriers using the B3LYP functional increase with the number of atoms in the molecule. Nonetheless, for relatively small molecules, the reliability of B3LYP is sufficient to show the overall trend in those reactions. Moreover, the TS predicted by B3LYP of those reactions has a precision comparable to more computationally expensive methods [66]. Given our focus on discerning overall trends in activation barriers, we have elected to adhere to the B3LYP/6‐311++G** level of theory primarily due to its favorable cost–benefit ratio compared to other computationally expensive methodologies. The R‐GPRI values were obtained using a modified version of the original GPRI software [13, 15, 18] which is available upon request.

## 4 Results

**FIGURE 1:**
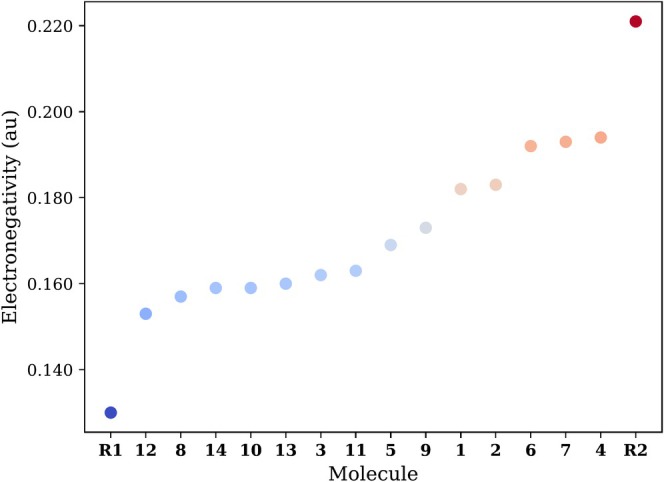
Mulliken electronegativity values in atomic units (au) for nitrogen heteroarenes (**1**−**14**), •*i*‐Pr (**R1**), and •CF_3_ (**R2**). The color scale ranges from blue (lowest) to red (highest).

Figure 1 shows the electronegativity values computed using Equation (6) for the 14 nitrogen heteroarenes (**1**−**14**) and two radicals, •*i*‐Pr (**R1**) and •CF_3_ (**R2**) under study, see Table S1 for the electronegativity values and Scheme 2 for the molecular structures. Overall, •*i*‐Pr has the lowest electronegativity and •CF_3_ the highest compared to nitrogen heteroarenes and radicals in this study. Hence, •CF_3_ is more likely to accept electrons (acting as an electrophilic radical), while ˙*i*‐Pr is most likely to donate electrons (behaving as a nucleophilic radical) [46].

**FIGURE 2:**
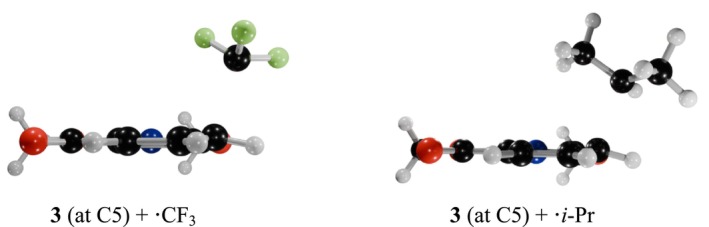
Transition state structures for the Reaction **3** (at C5) with •CF_3_ (left‐hand side), and for the Reaction **3** (at C5) + •*i*‐Pr (right‐hand side), calculated at the B3LYP/6‐311++G** level of theory.

Table 1 shows the calculated enthalpy activation barriers, ${\Delta H}_{298\;K}^{‡}$, for each reaction between the five likely reactive carbons labeled as C2–C6 in **1**–**14** (see Scheme 2) and the radicals •CF_3_, and •*i*‐Pr. A total of 140 TSs were computed to calculate the ${\Delta H}_{298\;K}^{‡}$. These TSs concord with the geometry adopted during a radical addition reaction [43, 70, 71], see Figure 2. The experimental yield ratio (EY) is written in parenthesis next to the ${\Delta H}_{298\;K}^{‡}$, and for the atoms where any product was experimentally isolated a “−” appears. Additionally, the condensed $f^{0}$, are listed for each of the atoms.

**TABLE 1:** The calculated activation barriers (${\Delta H}_{298\;K}^{‡}$) and the experimental yield ratio in parenthesis (EY) for the reactions between each of the likely reactive carbon in the nitrogen heteroarenes considered, see Scheme 2, and the two radicals: (a) •CF_3_, and (b) •*i*‐Pr.

Molecule	Atom	${\Delta H}_{298\;K}^{‡}$		$f^{0}$
		(a) •CF_3_ (EY)	(b) •*i*‐Pr (EY)	
**1**	C2	46.1 (−)	60.3 (−)	0.091
	C3	27.6 (0)	47.2 (−)	0.127
	C4	32.0 (−)	51.0 (−)	0.121
	C5	24.1 (1)	48.1 (−)	0.168
	C6	38.8 (−)	64.7 (−)	0.067
**2**	C2	45.0 (−)	59.0 (−)	0.093
	C3	36.5 (−)	65.1 (−)	0.070
	C4	31.2 (1)	50.7 (1)	0.111
	C5	25.5 (4)	45.0 (2.6)	0.169
	C6	28.8 (1)	48.7 (−)	0.136
**3**	C2	34.5 (−)	56.7 (−)	0.080
	C3	21.3 (1)	50.6 (0)	0.128
	C4	31.5 (−)	55.9 (−)	0.108
	C5	17.2 (7)	38.1 (1)	0.156
	C6	51.9 (−)	90.2 (−)	0.052
**4**	C2	46.8 (−)	52.3 (−)	0.088
	C3	29.1 (1)	40.5 (0)	0.126
	C4	34.5 (−)	47.9 (−)	0.116
	C5	26.0 (13)	34.1 (1)	0.161
	C6	50.3 (−)	58.1 (−)	0.054
**5**	C2	39.9 (−)	61.5 (−)	0.068
	C3	25.0 (1)	56.8 (−)	0.091
	C4	51.9 (−)	82.3 (−)	0.062
	C5	20.3 (1.7)	42.7 (−)	0.156
	C6	29.4 (−)	53.4 (−)	0.096
**6**	C2	45.3 (−)	50.8 (−)	0.087
	C3	31.1 (−)	46.3 (−)	0.108
	C4	32.7 (0)	38.2 (−)	0.112
	C5	50.0 (−)	60.5 (−)	0.089
	C6	27.7 (1)	31.4 (−)	0.114
**7**	C2	46.7 (−)	54.8 (−)	0.054
	C3	48.2 (−)	53.9 (−)	0.085
	C4	31.4 (0)	38.8 (1)	0.117
	C5	32.1 (−)	50.2 (−)	0.127
	C6	25.6 (1)	33.6 (2.3)	0.174
**8**	C2	33.1 (−)	67.2 (−)	0.080
	C3	25.9 (−)	53.2 (−)	0.119
	C4	29.8 (1)	58.2 (−)	0.149
	C5	41.9 (−)	66.4 (−)	0.066
	C6	26.2 (1.5)	51.2 (−)	0.129
**9**	C2	36.1 (−)	61.8 (−)	0.068
	C3	40.3 (−)	67.9 (−)	0.085
	C4	27.9 (1)	54.3 (1)	0.105
	C5	27.9 (−)	58.4 (−)	0.139
	C6	23.6 (1.1)	43.2 (10)	0.173
**10**	C2	23.4 (1.6)	54.4 (−)	0.115
	C3	31.5 (−)	75.6 (−)	0.071
	C4	35.2 (−)	74.8 (−)	0.033
	C5	24.6 (−)	63.9 (−)	0.125
	C6	22.9 (1)	54.3 (−)	0.134
**11**	C2	22.9 (1.4)	48.9 (1)	0.127
	C3	41.4 (−)	62.2 (−)	0.061
	C4	35.7 (−)	57.7 (−)	0.064
	C5	23.6 (−)	43.6 (−)	0.105
	C6	23.1 (1)	51.4 (0)	0.141
**12**	C2	23.0 (2.1)	49.2 (−)	0.155
	C3	44.6 (−)	72.8 (−)	0.060
	C4	24.0 (1)	45.4 (−)	0.137
	C5	44.6 (−)	68.4 (−)	0.057
	C6	23.8 (1.3)	47.0 (−)	0.137
**13**	C2	44.2 (−)	65.5 (−)	0.049
	C3	36.3 (−)	73.1 (−)	0.059
	C4	29.9 (1)	54.0 (−)	0.153
	C5	26.8 (−)	55.9 (−)	0.182
	C6	25.0 (4)	47.7 (−)	0.135
**14**	C2	27.3 (5)	46.0 (−)	0.134
	C3	42.9 (−)	68.4 (−)	0.062
	C4	40.4 (−)	69.0 (−)	0.073
	C5	27.5 (−)	59.4 (−)	0.121
	C6	25.0 (1)	50.2 (−)	0.153

*Note:* All data were calculated at 298 K using the B3LYP/6‐311++G** level of theory and reported in kJ/mol. A “−” indicates cases where no product was isolated experimentally. The condensed radical Fukui function, $f^{0}$, calculated using Equation (5) and the Hirshfeld population scheme of each of the carbon atoms considered in **1**–**14**.

Table 1 shows that reactions between nitrogen heteroarenes and •CF_3_ exhibit significantly lower ${\Delta H}_{298\;K}^{‡}$ values compared to those with •*i*‐Pr. The higher activation enthalpies of ˙*i*‐Pr justify the absence of experimentally isolated products in most cases.

Table 2 shows the performance of R‐GPRI and RFF in identifying the first and second most reactive atoms in Compounds **1**−**14**. The evaluation is based on available experimental data (*Exp*), represented by the EY ratio, and calculated enthalpy activation barrier, ${\Delta H}_{298\;K}^{‡}$, denoted as *Cal*. In the context of R‐GPRI, the most reactive atom is identified as the one most prevalent in the black box (electron transfer controlled, e^−^T), pink box (strong electron transfer controlled, Se^−^T), or their combination ERRC in the RTT analysis, see Tables S2–S29. For RFF, the most reactive atom is determined as the one with the highest RFF value.

**TABLE 2:** The performance of condensed R‐GPRI and condensed RFF in identifying susceptible atoms in nitrogen heteroarenes (**1**−**14**) to undergo a radical addition reaction is compared with calculated ${\Delta H}_{298\;K}^{‡}$ values (*Cal*), and experimental data (*Exp*).

Molecule	Radical	Product			R‐GPRI							
			Most reactive		e^−^T		Se^−^T		ERRC		$f^{0}$	
			*Cal*	*Exp*	*Cal*	*Exp*	*Cal*	*Exp*	*Cal*	*Exp*	*Cal*	*Exp*
**1**	•CF_3_	First	C5	C5	✓	✓	✓	✓	✓	✓	✓	✓
		Second	C3	−	✓	−	✓	−	✓	−	✓	−
	•*i*‐Pr	First	C3,5	−	✓	−	✓	−	✓	−	✓	−
		Second	C4	−	!	−	⨯	−	!	−	✓	−
**2**	•CF_3_	First	C5	C5	✓	✓	✓	✓	✓	✓	✓	✓
		Second	C6	C6	✓	✓	✓	✓	✓	✓	✓	✓
	•*i*‐Pr	First	C5	C5	✓	✓	✓	✓	✓	✓	✓	✓
		Second	C6	C4	✓	⨯	✓	!	✓	!	✓	⨯
**3**	•CF_3_	First	C5	C5	✓	✓	✓	✓	✓	✓	✓	✓
		Second	C3	C3	✓	✓	✓	✓	✓	✓	✓	✓
	•*i*‐Pr	First	C5	C5	✓	✓	✓	✓	✓	✓	✓	✓
		Second	C3	−	✓	−	✓	−	✓	−	✓	−
**4**	•CF_3_	First	C5	C5	✓	✓	✓	✓	✓	✓	✓	✓
		Second	C3	C3	✓	✓	✓	✓	✓	✓	✓	✓
	•*i*‐Pr	First	C5	C5	✓	✓	✓	✓	✓	✓	✓	✓
		Second	C3	−	✓	−	✓	−	✓	−	✓	−
**5**	•CF_3_	First	C5	C5	✓	✓	✓	✓	✓	✓	✓	✓
		Second	C3	C3	✓	✓	⨯	⨯	✓	✓	⨯	⨯
	•*i*‐Pr	First	C5	−	✓	−	✓	−	✓	−	✓	−
		Second	C6	−	✓	−	⨯	−	✓	−	✓	−
**6**	•CF_3_	First	C6	C6	!	!	✓	✓	✓	✓	✓	✓
		Second	C3,4	−	✓	−	✓	−	✓	−	✓	−
	•*i*‐Pr	First	C6	−	✓	−	⨯	−	✓	−	✓	−
		Second	C4	−	✓	−	✓	−	✓	−	✓	−
**7**	•CF_3_	First	C6	C6	✓	✓	✓	✓	✓	✓	✓	✓
		Second	C4,5	−	✓	−	✓	−	✓	−	✓	−
	•*i*‐Pr	First	C6	C6	✓	✓	✓	✓	✓	✓	✓	✓
		Second	C4	C4	✓	✓	⨯	⨯	!	!	⨯	⨯
**8**	•CF_3_	First	C3,6	C6	✓*	✓*	✓*	✓*	✓*	✓*	✓*	✓*
		Second	C4	C4	✓*	✓*	✓*	✓*	✓*	✓*	✓*	✓*
	•*i*‐Pr	First	C6,3	−	✓*	−	✓*	−	✓*	−	✓*	−
		Second	C4	−	✓*	−	✓*	−	✓*	−	✓*	−
**9**	•CF_3_	First	C6	C6	✓	✓	✓	✓	✓	✓	✓	✓
		Second	C4,5	C4	✓	⨯	✓	!	✓	!	✓	⨯
	•*i*‐Pr	First	C6	C6	✓	✓	✓	✓	✓	✓	✓	✓
		Second	C4	C4	!	!	!	!	!	!	⨯	⨯
**10**	•CF_3_	First	C6,2,5	C2	${✓}^{‡}$	⨯	${✓}^{‡}$	⨯	${✓}^{‡}$	⨯	${✓}^{‡}$	⨯
		Second	C6,2,5	C6	${✓}^{‡}$	✓	${✓}^{‡}$	⨯	${✓}^{‡}$	✓	${✓}^{‡}$	⨯
	•*i*‐Pr	First	C6	−	✓	−	!	−	✓	−	✓	−
		Second	C2	−	✓	−	⨯	−	✓	−	⨯	−
**11**	•CF_3_	First	C6,2,5	C2	${✓}^{‡}$	✓*	${✓}^{‡}$	✓*	${✓}^{‡}$	✓*	${✓}^{‡}$	✓*
		Second	C6,2,5	C6	${✓}^{‡}$	✓*	${✓}^{‡}$	✓*	${✓}^{‡}$	✓*	${✓}^{‡}$	✓*
	•*i*‐Pr	First	C5	C2	⨯	⨯	⨯	!	⨯	!	⨯	⨯
		Second	C2	−	✓	−	✓	−	✓	−	✓	−
**12**	•CF_3_	First	C2	C2	✓	✓	✓	✓	✓	✓	✓	✓
		Second	C6,4	C6, 4	✓	✓	✓	✓	✓	✓	✓	✓
	•*i*‐Pr	First	C4,6	−	✓*	−	✓*	−	✓*	−	✓*	−
		Second	C2	−	✓*	−	✓*	−	✓*	−	✓*	−
**13**	•CF_3_	First	C6,5	C6	✓	⨯	✓	!	✓	!	✓	⨯
		Second	C4	C4	✓	✓	✓	✓	✓	✓	✓	✓
	•*i*‐Pr	First	C6	−	!	−	⨯	−	!	−	✓*	−
		Second	C4,5	−	✓	−	✓	−	✓	−	✓*	−
**14**	•CF_3_	First	C6	C2	✓	⨯	✓	⨯	✓	⨯	✓	⨯
		Second	C2,5	C6	✓	!	✓	⨯	✓	!	✓	⨯
	•*i*‐Pr	First	C2	−	✓*	−	✓*	−	✓*	−	✓*	−
		Second	C6	−	✓*	−	✓*	−	✓*	−	✓*	−

*Note:* The “most reactive” column lists the most reactive atom for each method, a comma separating two atoms indicates activation barrier differences of less than 2 kJ/mol, $|{\Delta \Delta H}_{298\;K}^{‡}|\leq$ 2 kJ/mol or experimental yield ratios less than 0.2. In this case, the most reactive calculated/experimental atoms are listed from left to right from the values listed in Table 1. Three reactivity conditions were explored in R‐GPRI: electron transfer (e^−^T), strong electron transfer (Se^−^T), and a combination of both (ERRC). Symbols include “✓” for correct regioselectivity, “✓*” for inverted regioselectivity, ${✓}^{‡}$ for similar ${\Delta H}_{298\;K}^{‡}$, “!” when the most reactive atom appears at least in one cell, and an “⨯” is assigned when models fail. A “−” denotes instances where no product was isolated experimentally.

For each model, the following symbols are used to indicate its performance in predicting the first and second most reactive atoms relative to *Exp* and *Cal* results:

- ✓ The atom that appears most frequently in the respective RTT region or the atom with the highest RFF value.
- ${✓}^{‡}$ The most frequently occurring atom in the RTT or the highest RFF value that does not match the experimental or calculated data but is close in value. Specifically, atoms with activation enthalpy differences $|{\Delta \Delta H}_{298\;K}^{‡}|$ ≤ 2 kJ/mol (~0.5 kcal/mol) or an EY ≤ 0.2 are considered equivalently reactive in both the R‐GPRI and RFF models. For instance, in Reactions **10** +•CF_3_ and **11 +** •CF_3_, three atoms fall within this $|{\Delta \Delta H}_{298\;K}^{‡}|$ range, indicating no clear preference among them.
- ✓* When the models predict the most reactive atom as the one with the second smallest activation barrier or the second largest EY (with $|{\Delta \Delta H}_{298\;K}^{‡}|$ > 2 kJ/mol or EY > 0.2 from the most reactive atom), this indicates inverted regioselectivity in both the R‐GPRI and RFF models.
- ! This situation occurs only for R‐GPRI when the most reactive atom appears in at least one cell of the corresponding RTT region but is not the most prevalent.
- ⨯ When the model fails to predict the most susceptible atom based on experimental or calculated methods. For GPRI, the atom does not appear in any cell of the corresponding RTT region, and for RFF, the most reactive atom (*Exp* or *Cal*) does not match the highest RFF values.
− When no experimentally isolated product could be obtained from the reaction.

**FIGURE 3:**
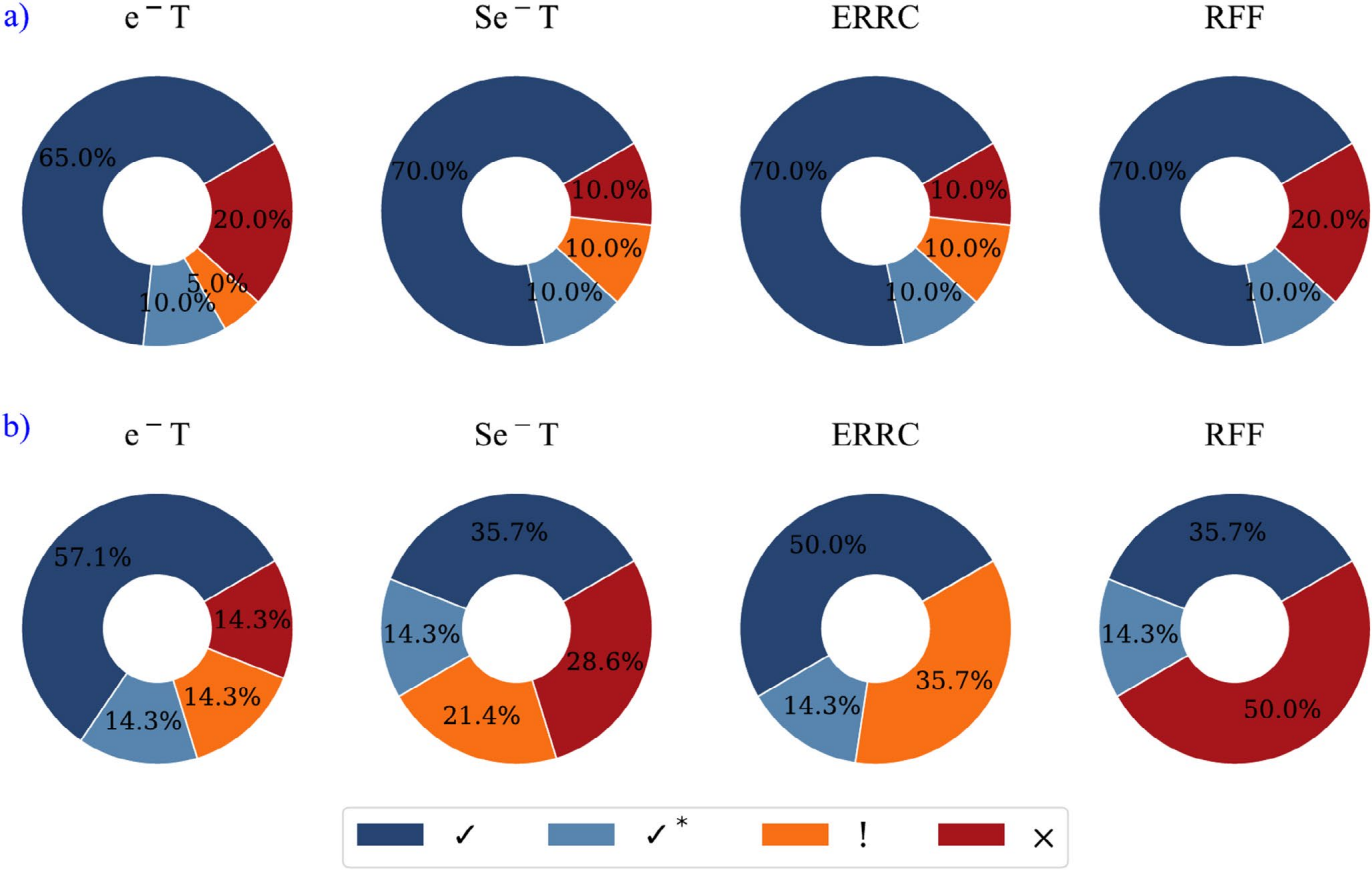
Performance of R‐GPRI, across three different reactivity situations: electron transfer, e^−^T, stronger electron transfer, Se^−^T, and the combination of both, ERRC, and RFF predicting the most reactive atoms in the reactions between the 14 nitrogen heteroarenes (**1**−**14**) and the two radicals for the *Exp* scenario. Rows (a) and (b) show model accuracy for the first and second most reactive atoms, respectively. Symbols: “✓” for correct regioselectivity, “✓*” for inverted regioselectivity, “!” when the most reactive atom appears at least in one cell but not the most prevalent atom, and a “⨯” is assigned when the most reactive atom is not predicted as reactive.

**FIGURE 4:**
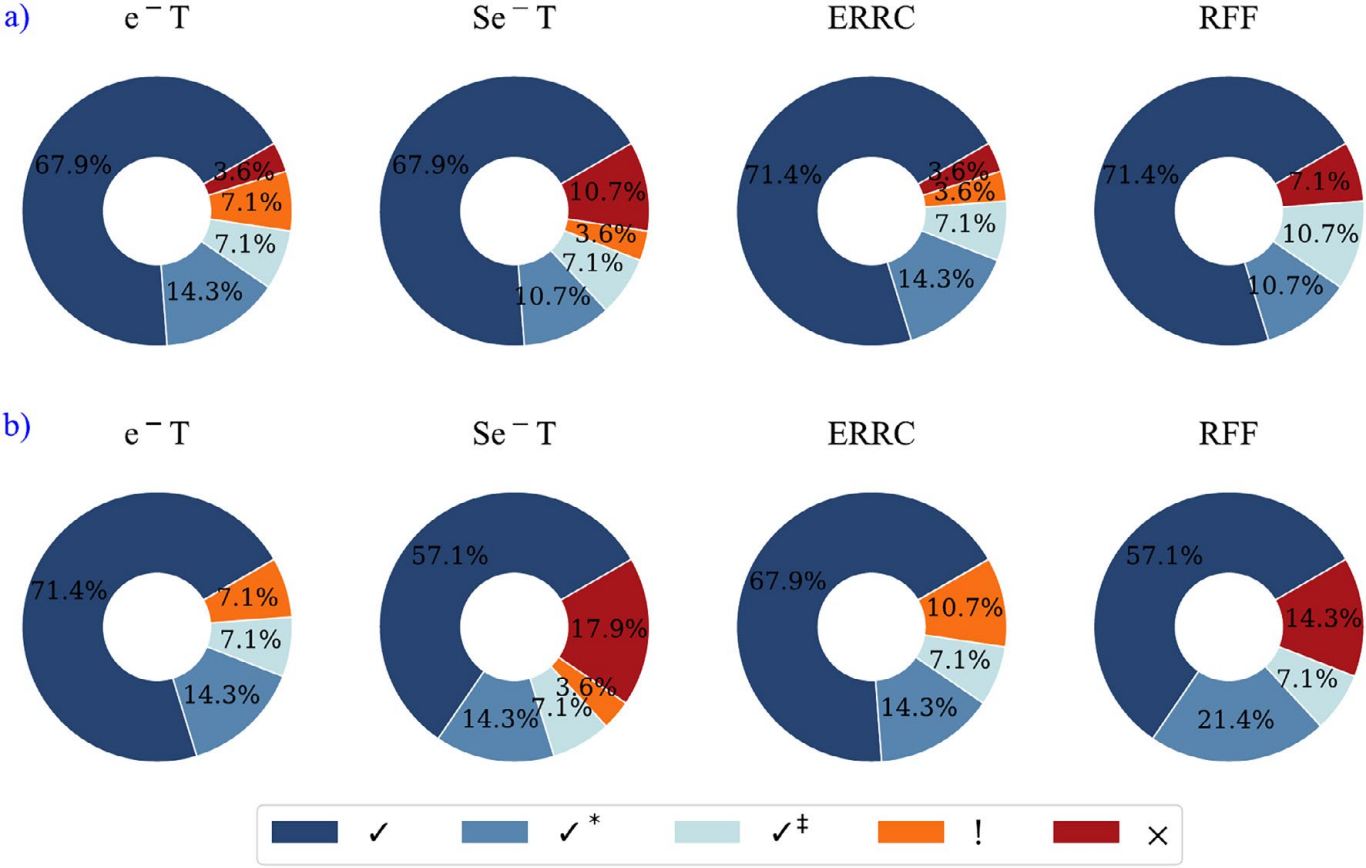
Performance of the R‐GPRI, across three different reactivity situations: electron transfer, e^−^T, stronger electron transfer, Se^−^T, and the combination of both, ERRC, and RFF predicting the most reactive atoms in the reactions between the 14 nitrogen heteroarenes (**1–14**) and the two radicals for the *Cal* scenario. Rows (a) and (b) show model accuracy for the first and second most reactive atoms, respectively. Symbols: “✓” for correct regioselectivity, “✓*” for inverted regioselectivity, “!” when the most reactive atom appears at least in one cell but not the most prevalent atom, and a “⨯” is assigned when the most reactive atom is not predicted as reactive.

Figures 3 and 4 summarize the findings from Table 2 regarding the performance of R‐GPRI (across three reactivity scenarios) and RFF in elucidating the two most reactive atoms in the reactions between nitrogen heteroarenes and radicals. These figures illustrate the predictive efficacy of both models in *Exp* (Figure 3) and *Cal* (Figure 4) scenarios, with rows (a) and (b) showing their performance in discerning the first and second most reactive sites, respectively.

Row (a) of Figure 3 displays that R‐GPRI correctly identified the most reactive atom (marked as ✓, ✓*, !) in 80% (e^−^T) and 90% (Se^−^T and ERRC) of cases, while RFF also achieved 80%, matching the R‐GPRI performance for e^−^T. Row (b) shows that R‐GPRI correctly identified the second most reactive atom in 85.7% (e^−^T), 71.4% (Se^−^T) and 100% (ERRC) of the studied cases, while RFF only correctly identified the second most reactive atom in 50% of the cases. This suggests that R‐GPRI performs better than RFF in identifying the second most reactive atom, while both models show comparable accuracy in predicting the first most reactive atom experimentally.

Since some reactions do not yield isolated products experimentally, we computed ${\Delta H}_{298\;K}^{‡}$ to identify the kinetically most reactive atoms (*Cal* in Table 2). Figure 4 summarizes the performance of R‐GPRI and RFF in identifying the first and second most reactive atoms based on the smallest calculated ${\Delta H}_{298\;K}^{‡}$. In row (a), R‐GPRI identified the most reactive atom (marked as ✓, ✓*, !) in 96.4% (e^−^T and ERRC) and 89.3% (Se^−^T) of the cases, while RFF achieved 92.1%. In row (b), R‐GPRI correctly identified the second most reactive atom in 100% (e^−^T and ERRC) and 82.1% (Se^−^T) of the cases, whereas RFF reached 85.7% of cases. Figure 4 highlights that both models effectively predict regioselectivity, with R‐GPRI slightly overperforming RFF in e^−^T and ERRC evaluations.

Figures 3 and 4 show that both RFF and R‐GPRI perform similarly in predicting the first most reactive atom in both *Exp* and *Cal* scenarios. However, R‐GPRI (e^−^T and ERRC) demonstrates greater reliability in identifying the second most reactive atom than RFF for both *Exp* and *Cal*.

In the experimental study by Baran et al. [10], the nitrogen heteroarenes were classified into two groups: conjugate (**1**–**7**) and innate (**8**–**14**). The reactivity of conjugate molecules is influenced by the attached groups on the aromatic ring (backbone), while the reactivity of innate heteroarenes is primarily influenced by the charge distribution on the backbone. Although the R‐GPRI and RFF models do not explicitly differentiate between conjugate and innate heteroarenes, we have categorized their performance according to Baran's classification. Figures S1 and S3 in the SM summarize the performance of the models in elucidating the two most reactive atoms in conjugate species (**1**–**7**), and Figures S2 and S4 in innate nitrogen heteroarenes (**8**–**14**), based on the outcomes detailed in Table 2. These figures compare the model performance under the *Exp* (Figures S1 and S2) and *Cal* (Figures S3 and S4) scenarios. This distinction highlights the strengths and limitations of both models in discerning the regioselectivity of these molecules. Overall, both models effectively elucidate the regioselectivity of conjugate species (**1**–**7**), though they encounter greater challenges with innate species (**8**–**14**).

Xiong et al. previously found that the condensed RFF computed using the Hirshfeld population scheme were ineffective in identifying the most reactive atoms susceptible to radical attacks across different heteroarenes [11]. However, our results displayed in Table 2, Figures 3, 4, and S1–S4 reveal that employing the Hirshfeld population scheme [12] and incorporating the atomic charges of hydrogen with their corresponding heavier atom (carbon) significantly enhanced the performance of the RFF in elucidating reactive sites. In previous work [14], we demonstrated that the R‐GPRI using the Hirshfeld population scheme correctly predicted the regioselectivity in 71.4% of the cases where the RFF failed. However, for the molecules examined in this study, both models demonstrate reliability in elucidating the first product. In contrast, the R‐GPRI outperforms the RFF in discerning the regioselectivity of the second product in these reactions. Also notice that the atoms that dominate the κ≈1 area of the RTTs, but are in few or none of the cells of the e^−^T, Se^−^T or the ERRC regions are never found to be the most or second most reactive by experimental outcomes nor calculated enthalpy. For example, C3 in **14** + •CF_3_ is the electrostatically preferred most reactive atom, see Table S28, but the R‐GPRI confirms the *Cal* and *Exp* findings that C3 is the least reactive under attack by a nucleophilic radical. Thus, our results confirm that these radical reactions are best described as electron transfer (orbital−controlled) and supports our choice of e^−^T and ERRC regions. Moreover, for reasonable choices of κ and ∆N within the RTT region of interest, approximately κ = −0.8, ΔN≈−0.3 for •CF_3_, and ∆N≈ 0.2 for •*i*‐Pr, the R‐GPRI values of each atom exhibit a linear correlation with their corresponding ${\Delta H}_{298\;K}^{‡}$, with R ^2^ ranging from 0.65 to 0.99, see Figures S5–S18.

## 5 Conclusions

We applied the condensed radical General‐Purpose Reactivity Indicator (R‐GPRI), a modified RFF that incorporates atomic charges (electrostatic effects), to identify the two most reactive atoms in 14 nitrogen heteroarenes reacting with the radicals •CF_3_, and •*i*‐Pr. The R‐GPRI and RFF outcomes were compared with available experimental data [10] and activation barriers (${\Delta H}_{298\;K}^{‡}$), calculated based on the TSs that represent the first step of 140 radical addition reactions, see Table 1. Our results show that R‐GPRI outperforms RFF in elucidating the second most reactive atom. However, both models exhibit similar performance in predicting the first most reactive site across all 14 nitrogen heteroarenes. Though R‐GPRI requires RTTs, which adding complexity, they also provide deeper insight into chemical reactivity across hard and soft conditions using the same number of calculations as RFF.

While RFF identifies the most reactive site, it struggles to predict the second, limiting its applicability in reactions that yield multiple products, such as radical C—H functionalization of nitrogen heteroarenes [10]. Notably, the performance of RFF improved when hydrogen charges were summed into their bonded heavy atoms.

The performance of R‐GPRI in elucidating both the first and second most reactive atoms is comparable using either the electron‐transfer (e^−^T) or ERRC regions of the RTTs. Therefore, we recommend using these regions to assess the reactivity of species susceptible to radical attacks. Given the success at predicting the second most reactive atom, it seems fair to conclude that the inclusion of atomic charges (electrostatic interactions) as a correction to RFF is important in predicting the most reactive atoms within R‐GPRI (e^−^T or ERRC). It is also important to note that the atoms that dominate the electrostatic regions of the RTTs but only have few or no entries in the cells of the e^−^T, Se^−^T, or the ERRC regions of the RTTs are never found to be reactive, for example, C3 in the reaction **14** + •CF_3_. These observations suggest that the reactions are Fukui/electron transfer controlled dominated, but it is necessary to include electrostatic contributions to elucidate the complete picture of reactivity.

## Supporting information


Data S1.

## Data Availability

The data that support the findings of this study are available from the corresponding author upon reasonable request.

## Appendix A Guidelines for Using the R‐GPRI

To apply the R‐GPRI in radical addition reactions, follow these steps:

1. Molecular optimization: optimize the neutral (or the state of interest of the) molecule of interest and compute single point energies for its anionic (or with an additional electron) and cationic (or with an electron removed) states.
2. Determine the nature of the attacking radical: use electronegativity values (Equation 6) to classify the attacking radical and thus to discern the appropriate R‐GPRI equation to use.
  - If the electronegativity of the radical is larger (more positive) than that of the molecule of interest, then the molecule of interest acts as an electrophile, and the appropriate R‐GPRI equation to use is Equation (1).
  - If the electronegativity of the radical is smaller (more negative) than that of the molecule of interest, then the molecule of interest acts as a nucleophile, and the appropriate R‐GPRI equation to use is Equation (2).
  - If no specific radical is being considered, then both R‐GPRI equations should be examined for a general electrophilic radical and nucleophilic radical attack.
3. Computing R‐GPRI values: calculate the proper R‐GPRI equation at different reactivity conditions (e.g., e^−^). The most reactive atom is identified as the one appearing most frequently in predefined areas; see Tables S2–S29.

## Appendix B Interpretation of R‐GPRI and the RTTs

The RTTs are a convenient and visually appealing way to display the results of the condensed R‐GPRI [13, 14, 16, 17, 18, 20, 21, 28, 29, 30]. They were introduced to display the results of the original GPRI [15] and have been used to display the results of the original GPRI and the R‐GPRI [13, 14, 16, 17, 18, 20, 21, 28, 29, 30]. The RTTs summarize the condensed R‐GPRI results for each atom under specific κ and ∆N values, which approximate the nature of the reaction. Since the R‐GPRI is based on modeling reaction energy, the most reactive atom is the one with the smallest R‐GPRI value (the “first choice” RTT), the atom with the second smallest value is the second most reactive (the “second choice” RTT), and so on. The motivation for displaying the results in the RTTs is that they can be read similarly to a phase diagram or a Pourbaix diagram, but with a different set of axes. In the spirit of how the phase and Pourbaix diagrams show the most stable matter phase or equilibrium phase respectively based on the lowest free energy at each point on the axes the “first choice” RTTs show the most reactive atom based on the lowest value of the condensed GPRI value at each point on the axes. Like a phase diagram or a Pourbaix diagram the RTTs do not explicitly include reaction rates and thus it is possible that at a point on the RTT that other atoms may have similar (but larger) values. Since the product of competing reactions are often yielded this motivates the “second choice” RTT, where at each point on the RTT is the atom with the second smallest value of the GPRI is placed. The axes of the RTTs are those of κ and ∆N that include information on the attacking molecule and the extent of electron transfer respectively. Just as in a phase diagram or a Pourbaix diagram, the greater the prevalence of an atom in the “first choice RTT” the more likely that atom is expected to be the most reactive atom, that is, the most prevalent atom is expected to be most reactive atom in most cases.

### Interpretation of κ and ∆N

In the R‐GPRI model determining the most reactive atoms is based on κ and ∆N pairs. As such interpreting these parameters is essential. ∆N is the amount of electron transfer in a reaction from (∆N<0) or to (∆N>0) the molecule of interest. The logical bounds for the extent of electron transfer for any reaction for an arbitrary attacking electrophile is −1≤ΔN≤0 and for an arbitrary attacking nucleophile is 0≤ΔN≤1. When the attacking molecule is known ∆N can be estimated [72],

(B1)
$$∆N=\frac{I+A}{2\left(I-A\right)}$$

where I and A are defined in Equations (7) and (8), respectively.

The values of κ are also chosen depending on the dominant reaction mechanism:

- κ>1 for strongly electrostatically‐controlled.
- κ≈1 for electrostatically‐controlled.
- −1<κ<1 for intermediate conditions (neither electrostatic nor electron transfer considerations dominate).
- κ≈−1 for electron‐transfer‐controlled.
- κ<−1 for strongly electron‐transfer‐controlled.

For specific reactivity conditions, κ can be estimated for the electrophilic‐ (erad) or nucleophilic‐ (nrad) radical employing Equations (B2) and (B3), respectively,

(B2)
$$\kappa \equiv q_{\text{erad}}^{\left(0\right)}+∆Nf_{\text{erad}}^{\left(0\right)}$$

(B3)
$$\;\kappa \equiv {-q}_{\text{nrad}}^{\left(0\right)}-∆Nf_{\text{nrad}}^{\left(0\right)}$$

### Application to Radical Addition Reactions

Radical reactions are generally electron‐transfer controlled, though with minimal transfer. Consequently, we expect that the optimal R‐GPRI conditions are κ≈−1 and ∆N≈0 [13, 14, 73, 74]. Using the interpretation given for κ and ∆N and the effective ranges of values used in previous work [13], we identified three key region of interest in R‐GPRI: e^−^T, Se^−^T, and ERRC.

The GPRI is derived using a truncated Taylor series expansion in terms of the external potential and number of electrons. The derivation assumes fixed nuclear positions and the attacking molecule is a point charge with Fukui function corrections. Therefore, R‐GPRI is most reliable for predicting the product when:

1. The attacking molecule is small (steric effects can be neglected).
2. The transition state is early (minimal structural change in the molecule at the transition state compared to the isolated geometry).
3. Higher‐order effects such as molecular polarization can be neglected.
