# Supplementary material for: Comparative Study of Predicting Radical C—H Functionalization Sites in Nitrogen Heteroarenes Using a Radical General‐Purpose Reactivity Indicator and the Radical Fukui Function
Source: J Comput Chem. 2025 May 19;46(14):e70130. doi: 10.1002/jcc.70130 (PMC12087272; doi:10.1002/jcc.70130)
Supplement: Supplementary file 1 — Data S1. [file JCC-46-0-s001.doc]

**Supplementary Material (SM)**

**Comparative Study of Predicting Radical C-H functionalization Sites in Nitrogen Heteroarenes Using a Radical General-Purpose Reactivity Indicator and the Radical Fukui Function**

Yoshio Barrera,^1^ James S. M. Anderson.^1*^

^1^Instituto de Química, Universidad Nacional Autónoma de México, Circuito Exterior, Ciudad Universitaria, Delegación Coyoacán, México City, C.P. 04510, México

*Corresponding author: James S. M. Anderson,

e-mail: james.anderson@iquimica.unam.mx

All calculations were carried out at the B3LYP/6-311++G** level of theory using the Gaussian 16 Rev. C01 package. All the molecules undergoing radical attack are shown in Scheme 2.

**Table S1.** Electronegativity values calculated using Equation (6),
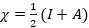
, for all of the chemical species considered. The values are listed from least electronegative (top) to most electronegative (bottom). All the units are in au.

| Molecule | Electronegativity |
| --- | --- |
| ⸱*i*-Pr (**R1**) | 0.130 |
| **12** | 0.153 |
| **8** | 0.157 |
| **14** | 0.159 |
| **10** | 0.159 |
| **13** | 0.160 |
| **3** | 0.162 |
| **11** | 0.163 |
| **5** | 0.169 |
| **9** | 0.173 |
| **1** | 0.182 |
| **2** | 0.183 |
| **6** | 0.192 |
| **7** | 0.193 |
| **4** | 0.194 |
| ⸱CF_3_ (**R2**) | 0.221 |

**
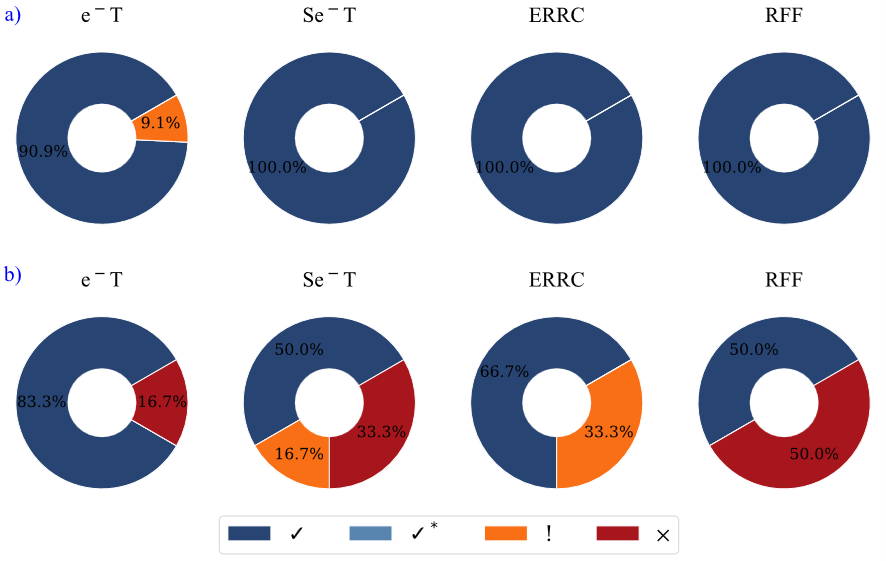
**

**Figure S1.** Performance of the R-GPRI, across three different reactivity situations: electron transfer, e^-^T, stronger electron transfer, Se^-^T, and the combination of both, ERRC, and RFF predicting the most reactive atoms in the reactions between **1**-**7** and the two radicals for the *Exp* scenarios. Rows a) and b) depict model accuracy for the prediction of the first and second product, respectively. “✓” for correct regioselectivity, “✓^*^” for inverted regioselectivity, “!” when the most reactive atom appears at least in one cell but not the most prevalent atom, and a “🗶” is assigned when the most reactive atom isn’t predicted to be reactive in any cell.

**
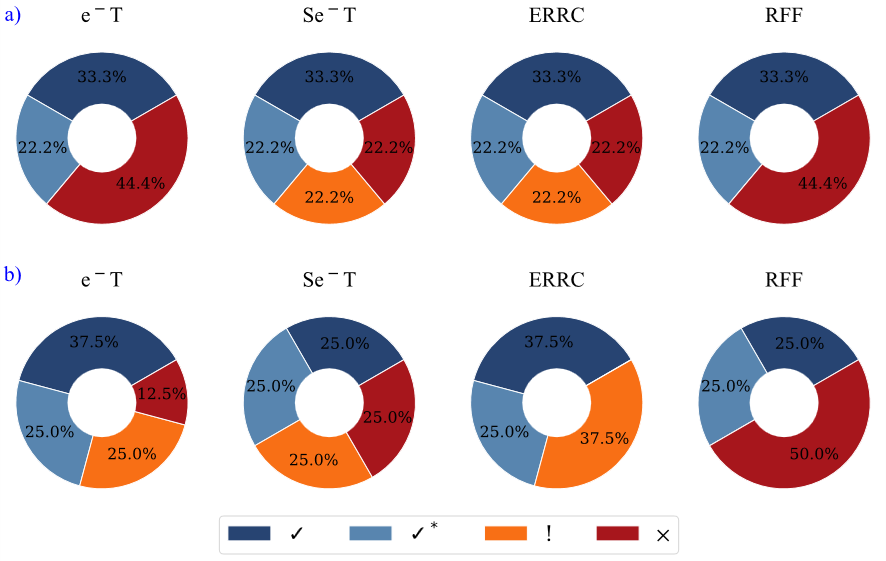
**

**Figure S2.** Performance of the R-GPRI, across three different reactivity situations: electron transfer, e^-^T, stronger electron transfer, Se^-^T, and the combination of both, ERRC, and RFF predicting the most reactive atoms in the reactions between **8**-**14** and the two radicals for the *Exp* scenarios. Rows a) and b) depict model accuracy for the prediction of the first and second product, respectively. “✓” for correct regioselectivity, “✓^*^” for inverted regioselectivity, “!” when the most reactive atom appears at least in one cell but not the most prevalent, and a “🗶” is assigned when the most reactive atom isn’t predicted to be reactive in any cell.


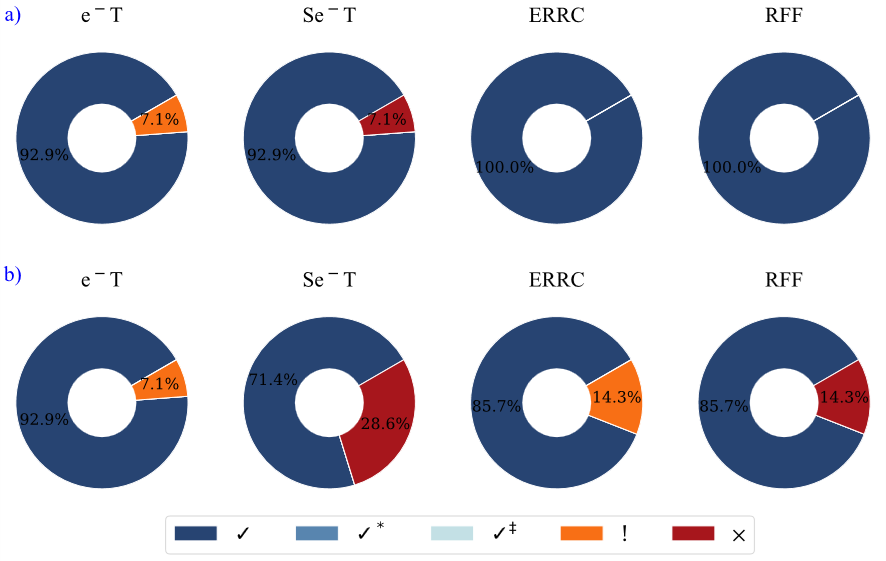


**Figure S3.** Performance of the R-GPRI, across three different reactivity situations: electron transfer, e^-^T, stronger electron transfer, Se^-^T, and the combination of both, ERRC, and RFF predicting the most reactive atoms in the reactions between **1**-**7** and the two radicals for the *Cal* scenarios. Rows a) and b) depict model accuracy of the prediction for first and second product, respectively. “✓” for correct regioselectivity, “✓^*^” for inverted regioselectivity,
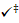
 for similar Δ
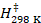
, “!” when the most reactive atom appears at least in one cell but not the most prevalent atom, and a “🗶” is assigned when the most reactive atom isn’t predicted to be reactive in any cell.


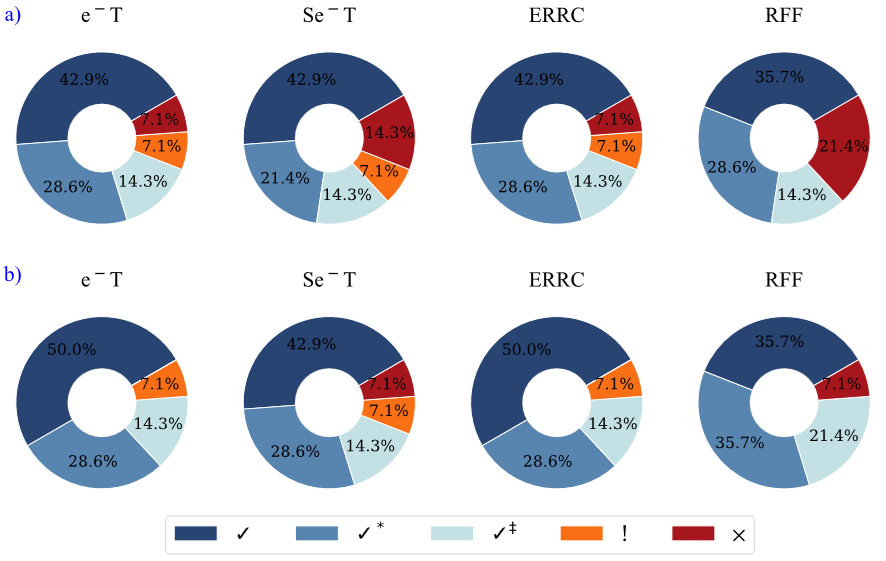


**Figure S4.** Performance of the R-GPRI, across three different reactivity situations: electron transfer, e^-^T, stronger electron transfer, Se^-^T, and the combination of both, ERRC, and RFF predicting the most reactive atoms in the reactions between **8**-**14** and the two radicals for the *Cal* scenarios. Rows a) and b) depict model accuracy for the prediction of the first and second product, respectively. “✓” for correct regioselectivity, “✓^*^” for inverted regioselectivity,
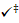
 for similar Δ
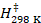
, “!” when the most reactive atom appears at least in one cell but not the most prevalent atom, and a “🗶” is assigned when the most reactive atom isn’t predicted to be reactive in any cell.

The symbol ⪅ is used when the enthalpies of activation differ by less than 2 kJ/mol and the symbol ≲ is used when the enthalpies of activation differ by less than 4 kJ/mol but differ by more than 2 kJ/mol. This is to indicate at the level of theory used that by activation barrier alone it is difficult to discern the kinetically most reactive atom kinetically second most reactive atom, etc, smaller than the margin of error.

**Table S2**. The predicted most and second most reactive carbon atom of the condensed R-GPRI,
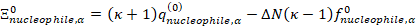
, for a given
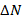
 and
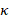
 using the Hirshfeld population scheme to compute the atomic charge,
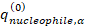
, and the atomic radical Fukui function,
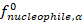
, with the contribution of the bonded hydrogen atom summed in, for the reaction: **1** + ⸱CF_3_.
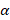
 represents the five likely reactive carbons in **1**, carbons labelled as C2-C6, see Scheme 2. Experimentally, the major product of this reaction is located at C5. No second product was isolated experimentally. The kinetically most reactive atoms as computed using the enthalpy of activation barriers, Δ
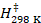
, are C5≲C3≲C4 (and C5<C4) with Δ
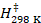
 of 24.15 kJ/mol, 27.63 kJ/mol and 32.05 kJ/mol, respectively.

a) First choice

| 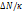 | 1 | 0.8 | | 0.6 | 0.4 | 0.2 | | 0 | -0.2 | -0.4 | | -0.6 | -0.8 | -1 | | -1.2 | -1.4 |
| --- | --- | --- | --- | --- | --- | --- | --- | --- | --- | --- | --- | --- | --- | --- | --- | --- | --- |
| -1 | 0.030 | -0.006 | | -0.042 | -0.079 | -0.116 | | -0.152 | -0.189 | -0.226 | | -0.262 | -0.299 | -0.336 | | -0.373 | -0.409 |
| -0.8 | 0.030 | 0.001 | | -0.029 | -0.059 | -0.089 | | -0.119 | -0.149 | -0.179 | | -0.209 | -0.239 | -0.269 | | -0.299 | -0.329 |
| -0.6 | 0.030 | 0.007 | | -0.015 | -0.039 | -0.062 | | -0.085 | -0.108 | -0.132 | | -0.155 | -0.178 | -0.201 | | -0.225 | -0.248 |
| -0.4 | 0.030 | 0.014 | | -0.002 | -0.018 | -0.035 | | -0.051 | -0.068 | -0.084 | | -0.101 | -0.118 | -0.134 | | -0.151 | -0.167 |
| -0.2 | 0.030 | 0.020 | | 0.011 | 0.001 | -0.008 | | -0.018 | -0.028 | -0.037 | | -0.047 | -0.057 | -0.067 | | -0.077 | -0.086 |
| 0.0 | 0.030 | 0.027 | | 0.024 | 0.021 | 0.018 | | 0.015 | 0.012 | 0.009 | | 0.006 | 0.003 |  | | -0.014 | -0.029 |
| 0.2 | 0.030 | 0.034 | | 0.038 | 0.041 | 0.045 | | 0.049 | 0.052 | 0.052 | | 0.051 | 0.039 | 0.026 | | 0.014 | 0.002 |
| C2 | | | C3 | | | | C4 | | | | C5 | | | | C6 | | |

b) Second choice

| 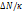 | 1 | 0.8 | | 0.6 | 0.4 | 0.2 | | 0 | -0.2 | -0.4 | | -0.6 | -0.8 | -1 | | -1.2 | -1.4 |
| --- | --- | --- | --- | --- | --- | --- | --- | --- | --- | --- | --- | --- | --- | --- | --- | --- | --- |
| -1 | 0.057 | 0.026 | | -0.004 | -0.036 | -0.067 | | -0.098 | -0.129 | -0.160 | | -0.191 | -0.222 | -0.253 | | -0.284 | -0.315 |
| -0.8 | 0.057 | 0.031 | | 0.005 | -0.020 | -0.046 | | -0.072 | -0.098 | -0.124 | | -0.150 | -0.176 | -0.202 | | -0.228 | -0.255 |
| -0.6 | 0.057 | 0.036 | | 0.015 | -0.005 | -0.026 | | -0.047 | -0.068 | -0.089 | | -0.110 | -0.131 | -0.152 | | -0.172 | -0.194 |
| -0.4 | 0.057 | 0.041 | | 0.025 | 0.009 | -0.006 | | -0.022 | -0.037 | -0.053 | | -0.069 | -0.085 | -0.101 | | -0.117 | -0.134 |
| -0.2 | 0.057 | 0.046 | | 0.035 | 0.024 | 0.014 | | 0.003 | -0.007 | -0.018 | | -0.029 | -0.039 | -0.050 | | -0.062 | -0.076 |
| 0.0 | 0.057 | 0.051 | | 0.045 | 0.039 | 0.034 | | 0.028 | 0.022 | 0.017 | | 0.011 | 0.006 |  | | -0.013 | -0.027 |
| 0.2 | 0.057 | 0.056 | | 0.055 | 0.055 | 0.054 | | 0.054 | 0.053 | 0.056 | | 0.052 | 0.046 | 0.036 | | 0.026 | 0.016 |
| C2 | | | C3 | | | | C4 | | | | C5 | | | | C6 | | |

**Table S3**. The predicted most and second most reactive carbon atom of the condensed R-GPRI,
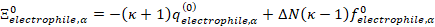
, for a given
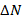
 and
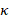
 using the Hirshfeld population scheme to compute the atomic charge,
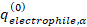
, and the atomic radical Fukui function,
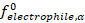
, with the contribution of the bonded atom summed in, for the reaction: **1** + ⸱*i*-Pr.
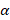
 represents the five likely reactive carbons in **1**, carbons labelled as C2-C6, see Scheme 2. No products were isolated experimentally. The kinetically most reactive atoms as computed using the enthalpy of activation barriers, Δ
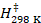
, are C3⪅C5≲C4 (and C3≲C4) with Δ
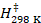
 of 47.21 kJ/mol, 48.06 kJ/mol and 51.01 kJ/mol, respectively.

a) First choice

| 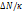 | 1 | 0.8 | | 0.6 | 0.4 | 0.2 | | 0 | -0.2 | -0.4 | | -0.6 | -0.8 | -1 | | -1.2 | -1.4 |
| --- | --- | --- | --- | --- | --- | --- | --- | --- | --- | --- | --- | --- | --- | --- | --- | --- | --- |
| -0.2 | -0.149 | -0.131 | | -0.113 | -0.096 | -0.078 | | -0.061 | -0.043 | -0.025 | | -0.008 | 0.009 | 0.026 | | 0.044 | 0.062 |
| 0.0 | -0.149 | -0.134 | | -0.119 | -0.104 | -0.089 | | -0.074 | -0.059 | -0.044 | | -0.029 | -0.014 |  | | 0.003 | 0.006 |
| 0.2 | -0.149 | -0.136 | | -0.124 | -0.112 | -0.100 | | -0.087 | -0.076 | -0.066 | | -0.059 | -0.063 | -0.067 | | -0.070 | -0.074 |
| 0.4 | -0.149 | -0.139 | | -0.130 | -0.120 | -0.111 | | -0.104 | -0.098 | -0.103 | | -0.113 | -0.124 | -0.134 | | -0.144 | -0.155 |
| 0.6 | -0.149 | -0.142 | | -0.135 | -0.128 | -0.125 | | -0.122 | -0.133 | -0.150 | | -0.167 | -0.184 | -0.201 | | -0.218 | -0.236 |
| 0.8 | -0.149 | -0.144 | | -0.140 | -0.139 | -0.140 | | -0.149 | -0.173 | -0.197 | | -0.221 | -0.245 | -0.269 | | -0.292 | -0.316 |
| 1.0 | -0.149 | -0.147 | | -0.146 | -0.150 | -0.154 | | -0.183 | -0.214 | -0.244 | | -0.275 | -0.305 | -0.336 | | -0.366 | -0.397 |
| C2 | | | C3 | | | | C4 | | | | C5 | | | | C6 | | |

b) Second choice

| 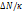 | 1 | 0.8 | | 0.6 | 0.4 | 0.2 | | 0 | -0.2 | -0.4 | | -0.6 | -0.8 | -1 | | -1.2 | -1.4 |
| --- | --- | --- | --- | --- | --- | --- | --- | --- | --- | --- | --- | --- | --- | --- | --- | --- | --- |
| -0.2 | -0.136 | -0.119 | | -0.101 | -0.084 | -0.067 | | -0.049 | -0.032 | -0.015 | | 0.001 | 0.019 | 0.036 | | 0.053 | 0.071 |
| 0.0 | -0.136 | -0.122 | | -0.109 | -0.095 | -0.081 | | -0.068 | -0.054 | -0.040 | | -0.027 | -0.013 |  | | 0.006 | 0.011 |
| 0.2 | -0.136 | -0.126 | | -0.116 | -0.106 | -0.096 | | -0.086 | -0.075 | -0.063 | | -0.056 | -0.052 | -0.050 | | -0.050 | -0.049 |
| 0.4 | -0.136 | -0.130 | | -0.123 | -0.117 | -0.110 | | -0.101 | -0.093 | -0.094 | | -0.095 | -0.096 | -0.101 | | -0.105 | -0.110 |
| 0.6 | -0.136 | -0.133 | | -0.130 | -0.128 | -0.121 | | -0.117 | -0.122 | -0.128 | | -0.134 | -0.142 | -0.152 | | -0.161 | -0.171 |
| 0.8 | -0.136 | -0.137 | | -0.138 | -0.136 | -0.132 | | -0.141 | -0.151 | -0.162 | | -0.173 | -0.188 | -0.202 | | -0.217 | -0.231 |
| 1.0 | -0.136 | -0.140 | | -0.145 | -0.144 | -0.153 | | -0.165 | -0.181 | -0.196 | | -0.214 | -0.233 | -0.253 | | -0.273 | -0.292 |
| C2 | | | C3 | | | | C4 | | | | C5 | | | | C6 | | |

**Table S4**. The predicted most and second most reactive carbon atom of the condensed R-GPRI,
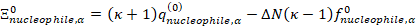
, for a given
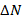
 and
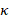
 using the Hirshfeld population scheme to compute the atomic charge,
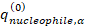
, and the atomic radical Fukui function,
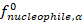
, with the contribution of the bonded hydrogen atom summed in, for the reaction: **2** + ⸱CF_3_.
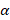
 represents the five likely reactive carbons in **2**, carbons labelled as C2-C6, see Scheme 2. Experimentally, the three major products of this reaction are located at C5, C4 and C6 with experimental yield ratio of 4:1:1. The kinetically most reactive atoms as computed using the enthalpy of activation barriers, Δ
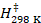
, are C5≲C6≲C4 (and C5<C4) with Δ
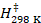
 of 25.50 kJ/mol, 28.77 kJ/mol and 31.17 kJ/mol, respectively.

a) First choice

| 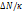 | 1 | 0.8 | | 0.6 | 0.4 | 0.2 | | 0 | -0.2 | -0.4 | | -0.6 | -0.8 | -1 | | -1.2 | -1.4 |
| --- | --- | --- | --- | --- | --- | --- | --- | --- | --- | --- | --- | --- | --- | --- | --- | --- | --- |
| -1 | 0.040 | 0.011 | | -0.027 | -0.066 | -0.105 | | -0.143 | -0.182 | -0.221 | | -0.259 | -0.298 | -0.337 | | -0.376 | -0.415 |
| -0.8 | 0.040 | 0.017 | | -0.014 | -0.046 | -0.078 | | -0.109 | -0.141 | -0.173 | | -0.205 | -0.237 | -0.269 | | -0.302 | -0.334 |
| -0.6 | 0.040 | 0.024 | | -0.001 | -0.026 | -0.051 | | -0.076 | -0.101 | -0.126 | | -0.151 | -0.177 | -0.202 | | -0.227 | -0.253 |
| -0.4 | 0.040 | 0.030 | | 0.012 | -0.006 | -0.024 | | -0.042 | -0.061 | -0.079 | | -0.097 | -0.116 | -0.134 | | -0.153 | -0.172 |
| -0.2 | 0.040 | 0.033 | | 0.026 | 0.014 | 0.003 | | -0.009 | -0.020 | -0.032 | | -0.044 | -0.055 | -0.067 | | -0.079 | -0.096 |
| 0.0 | 0.040 | 0.036 | | 0.032 | 0.028 | 0.024 | | 0.020 | 0.016 | 0.012 | | 0.008 | 0.004 |  | | -0.015 | -0.031 |
| 0.2 | 0.040 | 0.038 | | 0.037 | 0.036 | 0.035 | | 0.034 | 0.032 | 0.031 | | 0.030 | 0.029 | 0.027 | | 0.027 | 0.020 |
| C2 | | | C3 | | | | C4 | | | | C5 | | | | C6 | | |

b) Second choice

| 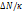 | 1 | 0.8 | | 0.6 | 0.4 | 0.2 | | 0 | -0.2 | -0.4 | | -0.6 | -0.8 | -1 | | -1.2 | -1.4 |
| --- | --- | --- | --- | --- | --- | --- | --- | --- | --- | --- | --- | --- | --- | --- | --- | --- | --- |
| -1 | 0.049 | 0.022 | | 0.004 | -0.020 | -0.049 | | -0.077 | -0.106 | -0.143 | | -0.186 | -0.228 | -0.271 | | -0.314 | -0.357 |
| -0.8 | 0.049 | 0.025 | | 0.010 | -0.007 | -0.031 | | -0.055 | -0.080 | -0.105 | | -0.142 | -0.179 | -0.217 | | -0.254 | -0.291 |
| -0.6 | 0.049 | 0.027 | | 0.015 | 0.003 | -0.013 | | -0.033 | -0.053 | -0.073 | | -0.099 | -0.131 | -0.162 | | -0.195 | -0.226 |
| -0.4 | 0.049 | 0.031 | | 0.021 | 0.011 | 0.001 | | -0.011 | -0.026 | -0.042 | | -0.057 | -0.082 | -0.108 | | -0.135 | -0.161 |
| -0.2 | 0.049 | 0.038 | | 0.026 | 0.019 | 0.013 | | 0.006 | -0.001 | -0.011 | | -0.022 | -0.033 | -0.054 | | -0.075 | -0.091 |
| 0.0 | 0.049 | 0.044 | | 0.039 | 0.034 | 0.029 | | 0.024 | 0.019 | 0.014 | | 0.009 | 0.004 |  | | -0.012 | -0.025 |
| 0.2 | 0.049 | 0.051 | | 0.053 | 0.055 | 0.056 | | 0.055 | 0.052 | 0.050 | | 0.048 | 0.045 | 0.037 | | 0.029 | 0.025 |
| C2 | | | C3 | | | | C4 | | | | C5 | | | | C6 | | |

**Table S5**. The predicted most and second most reactive carbon atom of the condensed R-GPRI,
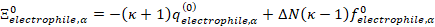
, for a given
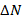
 and
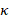
 using the Hirshfeld population scheme to compute the atomic charge,
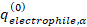
, and the atomic radical Fukui function,
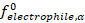
, with the contribution of the bonded hydrogen atom summed in, for the reaction: **2** + ⸱*i*-Pr.
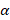
 represents the five likely reactive carbons in **2**, carbons labelled as C2-C6, see Scheme 2. Experimentally, the first two products are located at C5 and C4 with experimental yield ratio of 2.6:1. The kinetically most reactive atoms as computed using the enthalpy of activation barriers, Δ
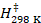
, are C5 and C6≲C4 with Δ
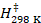
 of 43.03 kJ/mol, 48.67 kJ/mol, and 50.72 kJ/mol, respectively.

a) First choice

| 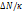 | 1 | 0.8 | | 0.6 | 0.4 | 0.2 | | 0 | -0.2 | -0.4 | | -0.6 | -0.8 | -1 | | -1.2 | -1.4 |
| --- | --- | --- | --- | --- | --- | --- | --- | --- | --- | --- | --- | --- | --- | --- | --- | --- | --- |
| -0.2 | -0.154 | -0.133 | | -0.113 | -0.091 | -0.071 | | -0.050 | -0.029 | -0.010 | | 0.005 | 0.021 | 0.027 | | 0.035 | 0.041 |
| 0.0 | -0.154 | -0.138 | | -0.123 | -0.108 | -0.093 | | -0.077 | -0.061 | -0.046 | | -0.030 | -0.015 |  | | 0.004 | 0.008 |
| 0.2 | -0.154 | -0.144 | | -0.134 | -0.124 | -0.114 | | -0.104 | -0.094 | -0.084 | | -0.074 | -0.065 | -0.067 | | -0.069 | -0.071 |
| 0.4 | -0.154 | -0.149 | | -0.145 | -0.140 | -0.136 | | -0.131 | -0.126 | -0.122 | | -0.117 | -0.126 | -0.134 | | -0.143 | -0.152 |
| 0.6 | -0.154 | -0.155 | | -0.156 | -0.156 | -0.158 | | -0.158 | -0.159 | -0.160 | | -0.171 | -0.187 | -0.202 | | -0.217 | -0.232 |
| 0.8 | -0.154 | -0.160 | | -0.166 | -0.173 | -0.179 | | -0.185 | -0.192 | -0.203 | | -0.225 | -0.247 | -0.269 | | -0.292 | -0.314 |
| 1.0 | -0.154 | -0.166 | | -0.177 | -0.189 | -0.201 | | -0.212 | -0.224 | -0.250 | | -0.279 | -0.308 | -0.337 | | -0.366 | -0.395 |
| C2 | | | C3 | | | | C4 | | | | C5 | | | | C6 | | |

b) Second choice

| 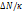 | 1 | 0.8 | | 0.6 | 0.4 | 0.2 | | 0 | -0.2 | -0.4 | | -0.6 | -0.8 | -1 | | -1.2 | -1.4 |
| --- | --- | --- | --- | --- | --- | --- | --- | --- | --- | --- | --- | --- | --- | --- | --- | --- | --- |
| -0.2 | -0.123 | -0.107 | | -0.091 | -0.075 | -0.059 | | -0.043 | -0.026 | -0.008 | | -0.012 | 0.021 | 0.037 | | 0.053 | 0.066 |
| 0.0 | -0.123 | -0.110 | | -0.098 | -0.086 | -0.073 | | -0.062 | -0.049 | -0.036 | | -0.024 | -0.012 |  | | 0.005 | 0.010 |
| 0.2 | -0.123 | -0.115 | | -0.106 | -0.097 | -0.088 | | -0.080 | -0.071 | -0.063 | | -0.064 | -0.064 | -0.054 | | -0.044 | -0.040 |
| 0.4 | -0.123 | -0.118 | | -0.113 | -0.108 | -0.103 | | -0.098 | -0.100 | -0.109 | | -0.117 | -0.113 | -0.108 | | -0.104 | -0.099 |
| 0.6 | -0.123 | -0.122 | | -0.121 | -0.119 | -0.118 | | -0.126 | -0.141 | -0.156 | | -0.161 | -0.162 | -0.162 | | -0.163 | -0.164 |
| 0.8 | -0.123 | -0.125 | | -0.128 | -0.131 | -0.137 | | -0.159 | -0.181 | -0.198 | | -0.204 | -0.210 | -0.217 | | -0.223 | -0.230 |
| 1.0 | -0.123 | -0.129 | | -0.135 | -0.142 | -0.164 | | -0.193 | -0.222 | -0.236 | | -0.248 | -0.259 | -0.271 | | -0.283 | -0.295 |
| C2 | | | C3 | | | | C4 | | | | C5 | | | | C6 | | |

**Table S6**. The predicted most and second most reactive carbon atom of the condensed R-GPRI,
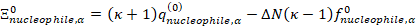
, for a given
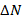
 and
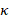
 using the Hirshfeld population scheme to compute the atomic charge,
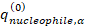
, and the atomic radical Fukui function,
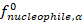
, with the contribution of the bonded hydrogen atom summed in, for the reaction: **3** + ⸱CF_3_.
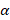
 represents the five likely reactive carbons in **3**, carbons labelled as C2-C6, see Scheme 2. Experimentally, the two major products of this reaction are located at C5 and C3 with experimental yield ratio of 7:1. The kinetically most reactive atoms as computed using the enthalpy of activation barriers, Δ
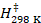
, are C5 and C3 with Δ
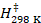
 of 17.17 kJ/mol and 21.30 kJ/mol, respectively.

a) First choice

| 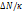 | 1 | 0.8 | | 0.6 | 0.4 | 0.2 | | 0 | -0.2 | -0.4 | | -0.6 | -0.8 | -1 | | -1.2 | -1.4 |
| --- | --- | --- | --- | --- | --- | --- | --- | --- | --- | --- | --- | --- | --- | --- | --- | --- | --- |
| -1 | 0.010 | -0.017 | | -0.050 | -0.082 | -0.116 | | -0.148 | -0.181 | -0.214 | | -0.247 | -0.280 | -0.312 | | -0.346 | -0.379 |
| -0.8 | 0.010 | -0.010 | | -0.037 | -0.064 | -0.090 | | -0.117 | -0.143 | -0.170 | | -0.197 | -0.224 | -0.250 | | -0.277 | -0.304 |
| -0.6 | 0.010 | -0.006 | | -0.025 | -0.045 | -0.065 | | -0.086 | -0.106 | -0.126 | | -0.147 | -0.167 | -0.187 | | -0.208 | -0.228 |
| -0.4 | 0.010 | -0.001 | | -0.012 | -0.026 | -0.040 | | -0.054 | -0.068 | -0.082 | | -0.097 | -0.111 | -0.125 | | -0.139 | -0.153 |
| -0.2 | 0.010 | 0.004 | | -0.002 | -0.008 | -0.015 | | -0.023 | -0.031 | -0.039 | | -0.046 | -0.054 | -0.062 | | -0.070 | -0.078 |
| 0.0 | 0.010 | 0.009 | | 0.008 | 0.007 | 0.006 | | 0.005 | 0.004 | 0.003 | | 0.002 | 0.001 |  | | -0.026 | -0.052 |
| 0.2 | 0.010 | 0.014 | | 0.018 | 0.022 | 0.026 | | 0.030 | 0.034 | 0.038 | | 0.041 | 0.036 | 0.020 | | -0.003 | -0.027 |
| C2 | | | C3 | | | | C4 | | | | C5 | | | | C6 | | |

b) Second choice

| 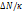 | 1 | 0.8 | | 0.6 | 0.4 | 0.2 | | 0 | -0.2 | -0.4 | | -0.6 | -0.8 | -1 | | -1.2 | -1.4 |
| --- | --- | --- | --- | --- | --- | --- | --- | --- | --- | --- | --- | --- | --- | --- | --- | --- | --- |
| -1 | 0.015 | -0.016 | | -0.042 | -0.069 | -0.095 | | -0.122 | -0.149 | -0.175 | | -0.202 | -0.228 | -0.255 | | -0.282 | -0.308 |
| -0.8 | 0.015 | -0.010 | | -0.032 | -0.053 | -0.075 | | -0.096 | -0.118 | -0.139 | | -0.161 | -0.182 | -0.204 | | -0.226 | -0.247 |
| -0.6 | 0.015 | -0.004 | | -0.022 | -0.038 | -0.055 | | -0.071 | -0.087 | -0.104 | | -0.120 | -0.136 | -0.153 | | -0.196 | -0.186 |
| -0.4 | 0.015 | 0.001 | | -0.012 | -0.023 | -0.034 | | -0.045 | -0.057 | -0.068 | | -0.079 | -0.090 | -0.102 | | -0.113 | -0.125 |
| -0.2 | 0.015 | 0.008 | | 0.000 | -0.008 | -0.014 | | -0.020 | -0.026 | -0.032 | | -0.038 | -0.044 | -0.051 | | -0.057 | -0.077 |
| 0.0 | 0.015 | 0.014 | | 0.012 | 0.011 | 0.009 | | 0.008 | 0.006 | 0.005 | | 0.003 | 0.001 |  | | -0.008 | -0.016 |
| 0.2 | 0.015 | 0.020 | | 0.025 | 0.029 | 0.034 | | 0.039 | 0.043 | 0.046 | | 0.042 | 0.044 | 0.032 | | 0.027 | 0.022 |
| C2 | | | C3 | | | | C4 | | | | C5 | | | | C6 | | |

**Table S7**. The predicted most and second most reactive carbon atom of the condensed R-GPRI,
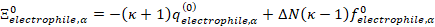
, for a given
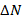
 and
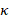
 using the Hirshfeld population scheme to compute the atomic charge,
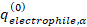
, and the atomic radical Fukui function,
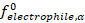
, with the contribution of the bonded hydrogen atom summed in, for the reaction: **3** + ⸱*i*-Pr.
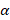
 represents the five likely reactive carbons in **3**, carbons labelled as C2-C6, see Scheme 2. Experimentally, the major product is located at C5. No second product was isolated experimentally. The kinetically most reactive atoms as computed using the enthalpy of activation barriers, Δ
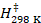
, are C5 and C3 with Δ
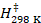
 of 38.14 kJ/mol and 50.59 kJ/mol, respectively.

a) First choice

| 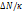 | 1 | 0.8 | | 0.6 | 0.4 | 0.2 | | 0 | -0.2 | -0.4 | | -0.6 | -0.8 | -1 | | -1.2 | -1.4 |
| --- | --- | --- | --- | --- | --- | --- | --- | --- | --- | --- | --- | --- | --- | --- | --- | --- | --- |
| -0.2 | -0.260 | -0.232 | | -0.203 | -0.175 | -0.147 | | -0.119 | -0.091 | -0.063 | | -0.035 | -0.007 | 0.020 | | 0.043 | 0.054 |
| 0.0 | -0.260 | -0.234 | | -0.208 | -0.182 | -0.156 | | -0.130 | -0.104 | -0.078 | | -0.052 | -0.026 |  | | 0.001 | 0.002 |
| 0.2 | -0.260 | -0.236 | | -0.212 | -0.188 | -0.164 | | -0.140 | -0.116 | -0.092 | | -0.068 | -0.058 | -0.062 | | -0.067 | -0.072 |
| 0.4 | -0.260 | -0.238 | | -0.216 | -0.194 | -0.172 | | -0.150 | -0.129 | -0.107 | | -0.103 | -0.114 | -0.125 | | -0.136 | -0.147 |
| 0.6 | -0.260 | -0.240 | | -0.220 | -0.200 | -0.181 | | -0.161 | -0.141 | -0.136 | | -0.153 | -0.170 | -0.187 | | -0.205 | -0.222 |
| 0.8 | -0.260 | -0.242 | | -0.224 | -0.207 | -0.189 | | -0.171 | -0.156 | -0.179 | | -0.203 | -0.226 | -0.250 | | -0.274 | -0.297 |
| 1.0 | -0.260 | -0.244 | | -0.228 | -0.213 | -0.197 | | -0.182 | -0.194 | -0.223 | | -0.253 | -0.283 | -0.312 | | -0.343 | -0.372 |
| C2 | | | C3 | | | | C4 | | | | C5 | | | | C6 | | |

b) Second choice

| 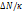 | 1 | 0.8 | | 0.6 | 0.4 | 0.2 | | 0 | -0.2 | -0.4 | | -0.6 | -0.8 | -1 | | -1.2 | -1.4 |
| --- | --- | --- | --- | --- | --- | --- | --- | --- | --- | --- | --- | --- | --- | --- | --- | --- | --- |
| -0.2 | -0.080 | -0.068 | | -0.057 | -0.046 | -0.035 | | -0.024 | -0.012 | -0.001 | | 0.010 | 0.020 | 0.032 | | 0.049 | 0.063 |
| 0.0 | -0.080 | -0.072 | | -0.064 | -0.056 | -0.048 | | -0.040 | -0.032 | -0.024 | | -0.016 | -0.008 |  | | 0.002 | 0.003 |
| 0.2 | -0.080 | -0.075 | | -0.070 | -0.065 | -0.060 | | -0.057 | -0.054 | -0.051 | | -0.053 | -0.047 | -0.051 | | -0.055 | -0.059 |
| 0.4 | -0.080 | -0.078 | | -0.076 | -0.075 | -0.077 | | -0.078 | -0.081 | -0.092 | | -0.085 | -0.092 | -0.102 | | -0.111 | -0.120 |
| 0.6 | -0.080 | -0.082 | | -0.083 | -0.088 | -0.094 | | -0.101 | -0.118 | -0.122 | | -0.124 | -0.138 | -0.153 | | -0.167 | -0.182 |
| 0.8 | -0.080 | -0.085 | | -0.091 | -0.101 | -0.111 | | -0.133 | -0.154 | -0.146 | | -0.165 | -0.184 | -0.204 | | -0.224 | -0.243 |
| 1.0 | -0.080 | -0.088 | | -0.100 | -0.114 | -0.134 | | -0.164 | -0.166 | -0.182 | | -0.206 | -0.231 | -0.255 | | -0.280 | -0.304 |
| C2 | | | C3 | | | | C4 | | | | C5 | | | | C6 | | |

**Table S8**. The predicted most and second most reactive carbon atom of the condensed R-GPRI,
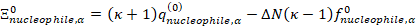
, for a given
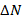
 and
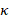
 using the Hirshfeld population scheme to compute the atomic charge,
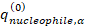
, and the atomic radical Fukui function,
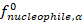
, with the contribution of the bonded hydrogen atom summed in, for the reaction: **4** + ⸱CF_3_.
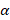
 represents the five likely reactive carbons in **4**, carbons labelled as C2-C6, see Scheme 2. Experimentally, the two major products of this reaction are located at C5 and C3 with experimental yield ratio of 13:1. The kinetically most reactive atoms as computed using the enthalpy of activation barriers, Δ
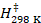
, are C5≲C3 with Δ
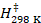
 of 26.04 kJ/mol and 29.07 kJ/mol, respectively.

a) First choice

| 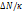 | 1 | 0.8 | | 0.6 | 0.4 | 0.2 | | 0 | -0.2 | -0.4 | | -0.6 | -0.8 | -1 | | -1.2 | -1.4 |
| --- | --- | --- | --- | --- | --- | --- | --- | --- | --- | --- | --- | --- | --- | --- | --- | --- | --- |
| -1 | 0.058 | 0.020 | | -0.017 | -0.055 | -0.093 | | -0.131 | -0.169 | -0.207 | | -0.245 | -0.283 | -0.321 | | -0.359 | -0.397 |
| -0.8 | 0.058 | 0.027 | | -0.004 | -0.035 | -0.067 | | -0.099 | -0.130 | -0.162 | | -0.193 | -0.225 | -0.256 | | -0.288 | -0.320 |
| -0.6 | 0.058 | 0.033 | | 0.008 | -0.016 | -0.041 | | -0.066 | -0.092 | -0.117 | | -0.142 | -0.167 | -0.192 | | -0.217 | -0.243 |
| -0.4 | 0.058 | 0.040 | | 0.021 | 0.002 | -0.016 | | -0.034 | -0.053 | -0.072 | | -0.091 | -0.109 | -0.128 | | -0.147 | -0.166 |
| -0.2 | 0.058 | 0.046 | | 0.034 | 0.021 | 0.009 | | -0.003 | -0.015 | -0.027 | | -0.039 | -0.051 | -0.064 | | -0.076 | -0.088 |
| 0.0 | 0.058 | 0.052 | | 0.046 | 0.041 | 0.035 | | 0.029 | 0.023 | 0.017 | | 0.011 | 0.005 |  | | -0.018 | -0.036 |
| 0.2 | 0.058 | 0.059 | | 0.059 | 0.060 | 0.060 | | 0.061 | 0.062 | 0.059 | | 0.052 | 0.037 | 0.021 | | 0.006 | -0.010 |
| C2 | | | C3 | | | | C4 | | | | C5 | | | | C6 | | |

b) Second choice

| 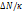 | 1 | 0.8 | | 0.6 | 0.4 | 0.2 | | 0 | -0.2 | -0.4 | | -0.6 | -0.8 | -1 | | -1.2 | -1.4 |
| --- | --- | --- | --- | --- | --- | --- | --- | --- | --- | --- | --- | --- | --- | --- | --- | --- | --- |
| -1 | 0.081 | 0.048 | | 0.015 | -0.018 | -0.051 | | -0.085 | -0.118 | -0.152 | | -0.185 | -0.218 | -0.252 | | -0.286 | -0.319 |
| -0.8 | 0.081 | 0.053 | | 0.025 | -0.003 | -0.031 | | -0.059 | -0.088 | -0.116 | | -0.145 | -0.173 | -0.201 | | -0.230 | -0.258 |
| -0.6 | 0.081 | 0.058 | | 0.035 | 0.011 | -0.011 | | -0.034 | -0.058 | -0.081 | | -0.104 | -0.128 | -0.151 | | -0.174 | -0.198 |
| -0.4 | 0.081 | 0.053 | | 0.045 | 0.027 | 0.008 | | -0.009 | -0.027 | -0.046 | | -0.064 | -0.082 | -0.100 | | -0.119 | -0.137 |
| -0.2 | 0.081 | 0.068 | | 0.055 | 0.042 | 0.028 | | 0.015 | 0.002 | -0.010 | | -0.023 | -0.037 | -0.050 | | -0.064 | -0.079 |
| 0.0 | 0.081 | 0.073 | | 0.065 | 0.057 | 0.049 | | 0.040 | 0.032 | 0.024 | | 0.016 | 0.008 |  | | -0.015 | -0.030 |
| 0.2 | 0.081 | 0.078 | | 0.075 | 0.072 | 0.069 | | 0.066 | 0.063 | 0.062 | | 0.056 | 0.047 | 0.035 | | 0.023 | 0.011 |
| C2 | | | C3 | | | | C4 | | | | C5 | | | | C6 | | |

**Table S9**. The predicted most and second most reactive carbon atom of the condensed R-GPRI,
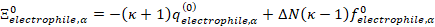
, for a given
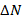
 and
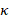
 using the Hirshfeld population scheme to compute the atomic charge,
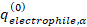
, and the atomic radical Fukui function,
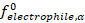
, with the contribution of the bonded hydrogen atom summed in, for the reaction: **4** + ⸱*i*-Pr.
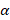
 represents the five likely reactive carbons in **4**, carbons labelled as C2-C6, see Scheme 2. Experimentally, the major product is located at C5. No second product was isolated experimentally. The kinetically most reactive atoms as computed using the enthalpy of activation barriers, Δ
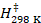
, are C5 and C3 with Δ
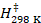
 of 34.08 kJ/mol and 40.47 kJ/mol, respectively.

a) First choice

| 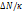 | 1 | 0.8 | | 0.6 | 0.4 | 0.2 | | 0 | -0.2 | -0.4 | | -0.6 | -0.8 | -1 | | -1.2 | -1.4 |
| --- | --- | --- | --- | --- | --- | --- | --- | --- | --- | --- | --- | --- | --- | --- | --- | --- | --- |
| -0.2 | -0.178 | -0.158 | | -0.138 | -0.118 | -0.098 | | -0.078 | -0.058 | -0.039 | | -0.018 | 0.001 | 0.021 | | 0.041 | 0.061 |
| 0.0 | -0.178 | -0.161 | | -0.143 | -0.125 | -0.107 | | -0.089 | -0.071 | -0.053 | | -0.035 | -0.017 |  | | 0.006 | 0.012 |
| 0.2 | -0.178 | -0.163 | | -0.147 | -0.131 | -0.115 | | -0.100 | -0.084 | -0.070 | | -0.063 | -0.063 | -0.064 | | -0.065 | -0.065 |
| 0.4 | -0.178 | -0.165 | | -0.151 | -0.138 | -0.124 | | -0.112 | -0.103 | -0.107 | | -0.114 | -0.121 | -0.128 | | -0.135 | -0.142 |
| 0.6 | -0.178 | -0.167 | | -0.155 | -0.144 | -0.134 | | -0.129 | -0.139 | -0.152 | | -0.165 | -0.179 | -0.192 | | -0.206 | -0.219 |
| 0.8 | -0.178 | -0.169 | | -0.160 | -0.150 | -0.148 | | -0.158 | -0.177 | -0.197 | | -0.217 | -0.237 | -0.256 | | -0.277 | -0.296 |
| 1.0 | -0.178 | -0.171 | | -0.164 | -0.160 | -0.163 | | -0.189 | -0.216 | -0.242 | | -0.268 | -0.294 | -0.321 | | -0.347 | -0.374 |
| C2 | | | C3 | | | | C4 | | | | C5 | | | | C6 | | |

b) Second choice

| 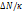 | 1 | 0.8 | | 0.6 | 0.4 | 0.2 | | 0 | -0.2 | -0.4 | | -0.6 | -0.8 | -1 | | -1.2 | -1.4 |
| --- | --- | --- | --- | --- | --- | --- | --- | --- | --- | --- | --- | --- | --- | --- | --- | --- | --- |
| -0.2 | -0.154 | -0.135 | | -0.116 | -0.097 | -0.078 | | -0.059 | -0.040 | -0.021 | | -0.002 | 0.016 | 0.035 | | 0.054 | 0.073 |
| 0.0 | -0.154 | -0.138 | | -0.123 | -0.108 | -0.092 | | -0.077 | -0.061 | -0.046 | | -0.030 | -0.015 |  | | 0.008 | 0.016 |
| 0.2 | -0.154 | -0.142 | | -0.130 | -0.118 | -0.106 | | -0.094 | -0.082 | -0.068 | | -0.060 | -0.053 | -0.050 | | -0.047 | -0.044 |
| 0.4 | -0.154 | -0.145 | | -0.137 | -0.128 | -0.120 | | -0.110 | -0.103 | -0.100 | | -0.098 | -0.099 | -0.100 | | -0.102 | -0.104 |
| 0.6 | -0.154 | -0.149 | | -0.144 | -0.139 | -0.133 | | -0.128 | -0.130 | -0.132 | | -0.137 | -0.144 | -0.151 | | -0.158 | -0.165 |
| 0.8 | -0.154 | -0.152 | | -0.151 | -0.150 | -0.145 | | -0.152 | -0.158 | -0.165 | | -0.177 | -0.189 | -0.201 | | -0.213 | -0.226 |
| 1.0 | -0.154 | -0.156 | | -0.158 | -0.157 | -0.163 | | -0.175 | -0.186 | -0.201 | | -0.218 | -0.235 | -0.252 | | -0.269 | -0.286 |
| C2 | | | C3 | | | | C4 | | | | C5 | | | | C6 | | |

**Table S10**. The predicted most and second most reactive carbon atom of the condensed R-GPRI,
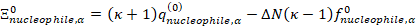
, for a given
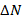
 and
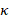
 using the Hirshfeld population scheme to compute the atomic charge,
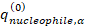
, and the atomic radical Fukui function,
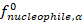
, with the contribution of the bonded hydrogen atom summed in, for the reaction: **5** + ⸱CF_3_.
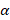
 represents the five likely reactive carbons in **5**, carbons labelled as C2-C6, see Scheme 2. Experimentally, the two major products of this reaction are located at C5 and C3 with experimental yield ratio of 1.7:1. The kinetically most reactive atoms as computed using the enthalpy of activation barriers, Δ
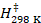
, are C5, C3 and C6 with Δ
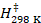
 of 20.34 kJ/mol, 25.02 kJ/mol, and 29.37 kJ/mol, respectively.

a) First choice

| 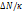 | 1 | 0.8 | | 0.6 | 0.4 | 0.2 | | 0 | -0.2 | -0.4 | | -0.6 | -0.8 | -1 | | -1.2 | -1.4 |
| --- | --- | --- | --- | --- | --- | --- | --- | --- | --- | --- | --- | --- | --- | --- | --- | --- | --- |
| -1 | -0.052 | -0.065 | | -0.078 | -0.091 | -0.121 | | -0.153 | -0.184 | -0.216 | | -0.248 | -0.279 | -0.311 | | -0.343 | -0.375 |
| -0.8 | -0.052 | -0.061 | | -0.071 | -0.080 | -0.096 | | -0.121 | -0.147 | -0.172 | | -0.198 | -0.223 | -0.249 | | -0.275 | -0.300 |
| -0.6 | -0.052 | -0.058 | | -0.063 | -0.069 | -0.075 | | -0.090 | -0.109 | -0.129 | | -0.148 | -0.279 | -0.187 | | -0.206 | -0.225 |
| -0.4 | -0.052 | -0.054 | | -0.056 | -0.058 | -0.060 | | -0.062 | -0.072 | -0.086 | | -0.098 | -0.111 | -0.124 | | -0.137 | -0.150 |
| -0.2 | -0.052 | -0.050 | | -0.049 | -0.047 | -0.046 | | -0.044 | -0.042 | -0.041 | | -0.048 | -0.055 | -0.062 | | -0.069 | -0.076 |
| 0.0 | -0.052 | -0.047 | | -0.041 | -0.037 | -0.031 | | -0.026 | -0.020 | -0.015 | | -0.010 | -0.005 |  | | -0.017 | -0.035 |
| 0.2 | -0.052 | -0.043 | | -0.034 | -0.026 | -0.017 | | -0.008 | 0.001 | 0.009 | | 0.018 | 0.027 | 0.024 | | 0.010 | -0.005 |
| C2 | | | C3 | | | | C4 | | | | C5 | | | | C6 | | |

b) Second choice

| 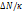 | 1 | 0.8 | | 0.6 | 0.4 | 0.2 | | 0 | -0.2 | -0.4 | | -0.6 | -0.8 | -1 | | -1.2 | -1.4 |
| --- | --- | --- | --- | --- | --- | --- | --- | --- | --- | --- | --- | --- | --- | --- | --- | --- | --- |
| -1 | 0.005 | -0.026 | | -0.057 | -0.089 | -0.104 | | -0.117 | -0.130 | -0.143 | | -0.156 | -0.169 | -0.193 | | -0.227 | -0.261 |
| -0.8 | 0.005 | -0.019 | | -0.045 | -0.070 | -0.089 | | -0.099 | -0.108 | -0.117 | | -0.127 | -0.136 | -0.154 | | -0.184 | -0.214 |
| -0.6 | 0.005 | -0.013 | | -0.032 | -0.052 | -0.071 | | -0.080 | -0.086 | -0.092 | | -0.097 | -0.103 | -0.115 | | -0.142 | -0.168 |
| -0.4 | 0.005 | -0.007 | | -0.020 | -0.033 | -0.046 | | -0.059 | -0.064 | -0.066 | | -0.068 | -0.070 | -0.077 | | -0.099 | -0.121 |
| -0.2 | 0.005 | -0.001 | | -0.007 | -0.015 | -0.021 | | -0.028 | -0.035 | -0.041 | | -0.039 | -0.038 | -0.038 | | -0.057 | -0.075 |
| 0.0 | 0.005 | 0.005 | | 0.004 | 0.003 | 0.003 | | 0.003 | 0.002 | 0.001 | | 0.001 | 0.001 |  | | -0.015 | -0.029 |
| 0.2 | 0.005 | 0.011 | | 0.017 | 0.022 | 0.028 | | 0.034 | 0.039 | 0.043 | | 0.038 | 0.032 | 0.027 | | 0.022 | 0.016 |
| C2 | | | C3 | | | | C4 | | | | C5 | | | | C6 | | |

**Table S11**. The predicted most and second most reactive carbon atom of the condensed R-GPRI,
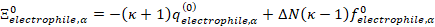
, for a given and using the Hirshfeld population scheme to compute the atomic charge, , and the atomic radical Fukui function, , with the contribution of the bonded hydrogen atom summed in, for the reaction: **5** + ⸱*i*-Pr. represents the five likely reactive carbons in **5**, carbons labelled as C2-C6, see Scheme 2. No products were isolated experimentally. The kinetically most reactive atoms as computed using the enthalpy of activation barriers, Δ, are C5 and C6≲C3 with Δ of 42.71 kJ/mol, 53.45 kJ/mol, and 56.83 kJ/mol, respectively.

a) First choice

|  | 1 | 0.8 | | 0.6 | 0.4 | 0.2 | | 0 | -0.2 | -0.4 | | -0.6 | -0.8 | -1 | | -1.2 | -1.4 |
| --- | --- | --- | --- | --- | --- | --- | --- | --- | --- | --- | --- | --- | --- | --- | --- | --- | --- |
| -0.2 | -0.173 | -0.153 | | -0.133 | -0.113 | -0.094 | | -0.074 | -0.054 | -0.034 | | -0.014 | 0.005 | 0.024 | | 0.035 | 0.033 |
| 0.0 | -0.173 | -0.156 | | -0.138 | -0.121 | -0.104 | | -0.086 | -0.069 | -0.052 | | -0.034 | -0.017 |  | | -0.005 | -0.010 |
| 0.2 | -0.173 | -0.158 | | -0.143 | -0.128 | -0.113 | | -0.099 | -0.084 | -0.070 | | -0.060 | -0.056 | -0.062 | | -0.068 | -0.074 |
| 0.4 | -0.173 | -0.161 | | -0.148 | -0.136 | -0.123 | | -0.111 | -0.104 | -0.097 | | -0.100 | -0.112 | -0.124 | | -0.136 | -0.148 |
| 0.6 | -0.173 | -0.163 | | -0.153 | -0.143 | -0.133 | | -0.130 | -0.127 | -0.132 | | -0.150 | -0.168 | -0.187 | | -0.205 | -0.223 |
| 0.8 | -0.173 | -0.166 | | -0.158 | -0.151 | -0.149 | | -0.150 | -0.151 | -0.176 | | -0.200 | -0.225 | -0.249 | | -0.274 | -0.298 |
| 1.0 | -0.173 | -0.168 | | -0.163 | -0.160 | -0.164 | | -0.169 | -0.189 | -0.219 | | -0.250 | -0.281 | -0.311 | | -0.342 | -0.373 |
| C2 | | | C3 | | | | C4 | | | | C5 | | | | C6 | | |

b) Second choice

|  | 1 | 0.8 | | 0.6 | 0.4 | 0.2 | | 0 | -0.2 | -0.4 | | -0.6 | -0.8 | -1 | | -1.2 | -1.4 |
| --- | --- | --- | --- | --- | --- | --- | --- | --- | --- | --- | --- | --- | --- | --- | --- | --- | --- |
| -0.2 | -0.146 | -0.127 | | -0.109 | -0.090 | -0.072 | | -0.053 | -0.035 | -0.016 | | 0.001 | 0.016 | 0.027 | | 0.038 | 0.049 |
| 0.0 | -0.146 | -0.131 | | -0.116 | -0.102 | -0.087 | | -0.073 | -0.058 | -0.043 | | -0.029 | -0.014 |  | | 0.001 | 0.001 |
| 0.2 | -0.146 | -0.135 | | -0.124 | -0.113 | -0.103 | | -0.092 | -0.081 | -0.069 | | -0.054 | -0.049 | -0.038 | | -0.045 | -0.054 |
| 0.4 | -0.146 | -0.139 | | -0.132 | -0.125 | -0.118 | | -0.111 | -0.099 | -0.088 | | -0.090 | -0.084 | -0.077 | | -0.085 | -0.098 |
| 0.6 | -0.146 | -0.143 | | -0.140 | -0.136 | -0.133 | | -0.124 | -0.114 | -0.124 | | -0.121 | -0.118 | -0.115 | | -0.125 | -0.142 |
| 0.8 | -0.146 | -0.146 | | -0.147 | -0.148 | -0.143 | | -0.136 | -0.151 | -0.151 | | -0.153 | -0.153 | -0.154 | | -0.165 | -0.185 |
| 1.0 | -0.146 | -0.150 | | -0.155 | -0.158 | -0.153 | | -0.158 | -0.174 | -0.178 | | -0.183 | -0.188 | -0.193 | | -0.206 | -0.229 |
| C2 | | | C3 | | | | C4 | | | | C5 | | | | C6 | | |

**Table S12**. The predicted most and second most reactive carbon atom of the condensed R-GPRI, , for a given and using the Hirshfeld population scheme to compute the atomic charge, , and the atomic radical Fukui function, , with the contribution of the bonded hydrogen atom summed in, for the reaction: **6** + ⸱CF_3_. represents the five likely reactive carbons in **6**, carbons labelled as C2-C6, see Scheme 2. Experimentally, the major product of this reaction is located at C6. No second product was isolated experimentally. The kinetically most reactive atoms as computed using the enthalpy of activation barriers, Δ, are C6≲C3⪅C4 with Δ of 27.69 kJ/mol, 31.10 kJ/mol, and 32.67 kJ/mol, respectively.

a) First choice

|  | 1 | 0.8 | | 0.6 | 0.4 | 0.2 | | 0 | -0.2 | -0.4 | | -0.6 | -0.8 | -1 | | -1.2 | -1.4 |
| --- | --- | --- | --- | --- | --- | --- | --- | --- | --- | --- | --- | --- | --- | --- | --- | --- | --- |
| -1 | 0.019 | -0.001 | | -0.021 | -0.040 | -0.060 | | -0.083 | -0.110 | -0.136 | | -0.163 | -0.190 | -0.229 | | -0.275 | -0.321 |
| -0.8 | 0.019 | 0.003 | | -0.014 | -0.030 | -0.046 | | -0.062 | -0.084 | -0.106 | | -0.128 | -0.151 | -0.183 | | -0.224 | -0.266 |
| -0.6 | 0.019 | 0.006 | | -0.007 | -0.019 | -0.032 | | -0.044 | -0.058 | -0.076 | | -0.094 | -0.112 | -0.137 | | -0.174 | -0.211 |
| -0.4 | 0.019 | 0.010 | | 0.001 | -0.008 | -0.017 | | -0.026 | -0.035 | -0.045 | | -0.059 | -0.073 | -0.092 | | -0.124 | -0.156 |
| -0.2 | 0.019 | 0.013 | | 0.008 | 0.002 | -0.003 | | -0.009 | -0.014 | -0.019 | | -0.025 | -0.034 | -0.046 | | -0.073 | -0.101 |
| 0.0 | 0.019 | 0.017 | | 0.015 | 0.013 | 0.011 | | 0.009 | 0.007 | 0.006 | | 0.004 | 0.002 |  | | -0.023 | -0.046 |
| 0.2 | 0.019 | 0.020 | | 0.022 | 0.024 | 0.025 | | 0.027 | 0.029 | 0.031 | | 0.032 | 0.034 | 0.035 | | 0.020 | 0.005 |
| C2 | | | C3 | | | | C4 | | | | C5 | | | | C6 | | |

b) Second choice

|  | 1 | 0.8 | | 0.6 | 0.4 | 0.2 | | 0 | -0.2 | -0.4 | | -0.6 | -0.8 | -1 | | -1.2 | -1.4 |
| --- | --- | --- | --- | --- | --- | --- | --- | --- | --- | --- | --- | --- | --- | --- | --- | --- | --- |
| -1 | 0.051 | 0.025 | | -0.002 | -0.029 | -0.056 | | -0.080 | -0.100 | -0.119 | | -0.151 | -0.188 | -0.225 | | -0.261 | -0.298 |
| -0.8 | 0.051 | 0.029 | | 0.006 | -0.016 | -0.039 | | -0.061 | -0.078 | -0.094 | | -0.115 | -0.147 | -0.180 | | -0.212 | -0.244 |
| -0.6 | 0.051 | 0.033 | | 0.015 | -0.003 | -0.021 | | -0.039 | -0.057 | -0.069 | | -0.082 | -0.107 | -0.135 | | -0.163 | -0.191 |
| -0.4 | 0.051 | 0.038 | | 0.024 | 0.010 | -0.004 | | -0.018 | -0.032 | -0.044 | | -0.053 | -0.066 | -0.090 | | -0.113 | -0.137 |
| -0.2 | 0.051 | 0.042 | | 0.032 | 0.023 | 0.013 | | 0.004 | -0.005 | -0.015 | | -0.024 | -0.030 | -0.045 | | -0.064 | -0.083 |
| 0.0 | 0.051 | 0.046 | | 0.041 | 0.036 | 0.031 | | 0.026 | 0.021 | 0.015 | | 0.010 | 0.005 |  | | -0.018 | -0.037 |
| 0.2 | 0.051 | 0.051 | | 0.050 | 0.049 | 0.048 | | 0.047 | 0.047 | 0.046 | | 0.045 | 0.044 | 0.036 | | 0.027 | 0.009 |
| C2 | | | C3 | | | | C4 | | | | C5 | | | | C6 | | |

**Table S13**. The predicted most and second most reactive carbon atom of the condensed R-GPRI, , for a given and using the Hirshfeld population scheme to compute the atomic charge, , and the atomic radical Fukui function, , with the contribution of the bonded hydrogen atom summed in, for the reaction: **6** + ⸱*i*-Pr. represents the five likely reactive carbons in **6**, carbons labelled as C2-C6, see Scheme 2. No products were isolated experimentally. The kinetically most reactive atoms as computed using the enthalpy of activation barriers, Δ, are C6, C4, and C3 with Δ of 31.40 kJ/mol, 38.20 kJ/mol, and 46.32 kJ/mol, respectively.

a) First choice

|  | 1 | 0.8 | | 0.6 | 0.4 | 0.2 | | 0 | -0.2 | -0.4 | | -0.6 | -0.8 | -1 | | -1.2 | -1.4 |
| --- | --- | --- | --- | --- | --- | --- | --- | --- | --- | --- | --- | --- | --- | --- | --- | --- | --- |
| -0.2 | -0.230 | -0.203 | | -0.175 | -0.148 | -0.120 | | -0.092 | -0.065 | -0.037 | | -0.009 | 0.013 | 0.035 | | 0.041 | 0.047 |
| 0.0 | -0.230 | -0.207 | | -0.184 | -0.161 | -0.138 | | -0.115 | -0.092 | -0.069 | | -0.046 | -0.023 |  | | 0.002 | 0.004 |
| 0.2 | -0.230 | -0.212 | | -0.193 | -0.175 | -0.157 | | -0.138 | -0.120 | -0.101 | | -0.083 | -0.064 | -0.046 | | -0.043 | -0.042 |
| 0.4 | -0.230 | -0.216 | | -0.203 | -0.189 | -0.175 | | -0.161 | -0.147 | -0.133 | | -0.119 | -0.105 | -0.092 | | -0.090 | -0.094 |
| 0.6 | -0.230 | -0.221 | | -0.212 | -0.202 | -0.193 | | -0.184 | -0.174 | -0.165 | | -0.156 | -0.147 | -0.137 | | -0.138 | -0.146 |
| 0.8 | -0.230 | -0.226 | | -0.221 | -0.216 | -0.211 | | -0.207 | -0.202 | -0.197 | | -0.192 | -0.188 | -0.183 | | -0.186 | -0.198 |
| 1.0 | -0.230 | -0.230 | | -0.230 | -0.230 | -0.230 | | -0.230 | -0.229 | -0.229 | | -0.229 | -0.229 | -0.229 | | -0.233 | -0.250 |
| C2 | | | C3 | | | | C4 | | | | C5 | | | | C6 | | |

b) Second choice

|  | 1 | 0.8 | | 0.6 | 0.4 | 0.2 | | 0 | -0.2 | -0.4 | | -0.6 | -0.8 | -1 | | -1.2 | -1.4 |
| --- | --- | --- | --- | --- | --- | --- | --- | --- | --- | --- | --- | --- | --- | --- | --- | --- | --- |
| -0.2 | -0.185 | -0.163 | | -0.141 | -0.119 | -0.097 | | -0.075 | -0.053 | -0.031 | | -0.009 | 0.018 | 0.036 | | 0.053 | 0.062 |
| 0.0 | -0.185 | -0.166 | | -0.148 | -0.129 | -0.111 | | -0.092 | -0.074 | -0.055 | | -0.037 | -0.018 |  | | 0.005 | 0.010 |
| 0.2 | -0.185 | -0.170 | | -0.155 | -0.140 | -0.125 | | -0.110 | -0.095 | -0.080 | | -0.065 | -0.055 | -0.045 | | -0.037 | -0.039 |
| 0.4 | -0.185 | -0.173 | | -0.162 | -0.150 | -0.139 | | -0.127 | -0.116 | -0.106 | | -0.101 | -0.095 | -0.090 | | -0.084 | -0.082 |
| 0.6 | -0.185 | -0.177 | | -0.169 | -0.161 | -0.153 | | -0.145 | -0.139 | -0.138 | | -0.137 | -0.136 | -0.135 | | -0.134 | -0.133 |
| 0.8 | -0.185 | -0.180 | | -0.176 | -0.171 | -0.167 | | -0.162 | -0.166 | -0.169 | | -0.173 | -0.176 | -0.180 | | -0.183 | -0.187 |
| 1.0 | -0.185 | -0.184 | | -0.183 | -0.182 | -0.181 | | -0.185 | -0.193 | -0.201 | | -0.209 | -0.217 | -0.225 | | -0.232 | -0.240 |
| C2 | | | C3 | | | | C4 | | | | C5 | | | | C6 | | |

**Table S14**. The predicted most and second most reactive carbon atom of the condensed R-GPRI, , for a given and using the Hirshfeld population scheme to compute the atomic charge, , and the atomic radical Fukui function, , with the contribution of the bonded hydrogen atom summed in, for the reaction: **7** + ⸱CF_3_. represents the five likely reactive carbons in **7**, carbons labelled as C2-C6, see Scheme 2. Experimentally, the major product of this reaction is located at C6. No second product was isolated experimentally. The kinetically most reactive atoms as computed using the enthalpy of activation barriers, Δ, are C6 and C4⪅C5 with Δ of 25.56 kJ/mol, 31.45 kJ/mol, and 32.08 kJ/mol, respectively.

a) First choice

|  | 1 | 0.8 | | 0.6 | 0.4 | 0.2 | | 0 | -0.2 | -0.4 | | -0.6 | -0.8 | -1 | | -1.2 | -1.4 |
| --- | --- | --- | --- | --- | --- | --- | --- | --- | --- | --- | --- | --- | --- | --- | --- | --- | --- |
| -1 | 0.010 | -0.008 | | -0.026 | -0.044 | -0.070 | | -0.100 | -0.131 | -0.182 | | -0.238 | -0.293 | -0.349 | | -0.405 | -0.460 |
| -0.8 | 0.010 | -0.004 | | -0.019 | -0.034 | -0.049 | | -0.075 | -0.101 | -0.133 | | -0.182 | -0.231 | -0.279 | | -0.328 | -0.376 |
| -0.6 | 0.010 | -0.001 | | -0.012 | -0.023 | -0.035 | | -0.050 | -0.070 | -0.091 | | -0.126 | -0.168 | -0.209 | | -0.251 | -0.293 |
| -0.4 | 0.010 | 0.002 | | -0.005 | -0.013 | -0.021 | | -0.029 | -0.040 | -0.055 | | -0.071 | -0.105 | -0.140 | | -0.174 | -0.209 |
| -0.2 | 0.010 | 0.006 | | 0.001 | -0.003 | -0.007 | | -0.012 | -0.016 | -0.021 | | -0.030 | -0.042 | -0.070 | | -0.098 | -0.125 |
| 0.0 | 0.010 | 0.009 | | 0.008 | 0.007 | 0.006 | | 0.005 | 0.004 | 0.003 | | 0.002 | 0.001 |  | | -0.021 | -0.041 |
| 0.2 | 0.010 | 0.013 | | 0.015 | 0.017 | 0.020 | | 0.022 | 0.024 | 0.027 | | 0.029 | 0.031 | 0.022 | | 0.005 | -0.011 |
| C2 | | | C3 | | | | C4 | | | | C5 | | | | C6 | | |

b) Second choice

|  | 1 | 0.8 | | 0.6 | 0.4 | 0.2 | | 0 | -0.2 | -0.4 | | -0.6 | -0.8 | -1 | | -1.2 | -1.4 |
| --- | --- | --- | --- | --- | --- | --- | --- | --- | --- | --- | --- | --- | --- | --- | --- | --- | --- |
| -1 | 0.053 | 0.022 | | -0.008 | -0.039 | -0.062 | | -0.080 | -0.126 | -0.162 | | -0.192 | -0.223 | -0.254 | | -0.284 | -0.315 |
| -0.8 | 0.053 | 0.027 | | 0.002 | -0.024 | -0.048 | | -0.063 | -0.085 | -0.126 | | -0.152 | -0.177 | -0.203 | | -0.229 | -0.254 |
| -0.6 | 0.053 | 0.032 | | 0.012 | -0.009 | -0.029 | | -0.046 | -0.057 | -0.084 | | -0.111 | -0.132 | -0.152 | | -0.173 | -0.197 |
| -0.4 | 0.053 | 0.038 | | 0.022 | 0.007 | -0.009 | | -0.024 | -0.037 | -0.044 | | -0.070 | -0.086 | -0.102 | | -0.117 | -0.141 |
| -0.2 | 0.053 | 0.043 | | 0.032 | 0.022 | 0.012 | | 0.001 | -0.009 | -0.020 | | -0.025 | -0.040 | -0.051 | | -0.066 | -0.085 |
| 0.0 | 0.053 | 0.048 | | 0.042 | 0.037 | 0.032 | | 0.027 | 0.021 | 0.016 | | 0.011 | 0.005 |  | | -0.019 | -0.037 |
| 0.2 | 0.053 | 0.053 | | 0.053 | 0.052 | 0.052 | | 0.052 | 0.052 | 0.051 | | 0.051 | 0.038 | 0.034 | | 0.036 | 0.028 |
| C2 | | | C3 | | | | C4 | | | | C5 | | | | C6 | | |

**Table S15**. The predicted most and second most reactive carbon atom of the condensed R-GPRI, , for a given and using the Hirshfeld population scheme to compute the atomic charge, , and the atomic radical Fukui function, , with the contribution of the bonded hydrogen atom summed in, for the reaction: **7** + ⸱*i*-Pr. represents the five likely reactive carbons in **7**, carbons labelled as C2-C6, see Scheme 2. Experimentally, the two major products of this reaction are located at C6 and C4 with experimental yield ratio of 2.3:1. The kinetically most reactive atoms as computed using the enthalpy of activation barriers, Δ, are C6 and C4 with Δ of 33.62 kJ/mol, and 38.82 kJ/mol, respectively.

a) First choice

|  | 1 | 0.8 | | 0.6 | 0.4 | 0.2 | | 0 | -0.2 | -0.4 | | -0.6 | -0.8 | -1 | | -1.2 | -1.4 |
| --- | --- | --- | --- | --- | --- | --- | --- | --- | --- | --- | --- | --- | --- | --- | --- | --- | --- |
| -0.2 | -0.207 | -0.180 | | -0.152 | -0.124 | -0.104 | | -0.083 | -0.062 | -0.041 | | -0.020 | 0.001 | 0.022 | | 0.038 | 0.043 |
| 0.0 | -0.207 | -0.187 | | -0.166 | -0.145 | -0.124 | | -0.104 | -0.083 | -0.062 | | -0.041 | -0.021 |  | | 0.001 | 0.002 |
| 0.2 | -0.207 | -0.194 | | -0.180 | -0.166 | -0.152 | | -0.139 | -0.125 | -0.111 | | -0.097 | -0.084 | -0.070 | | -0.056 | -0.050 |
| 0.4 | -0.207 | -0.200 | | -0.194 | -0.187 | -0.180 | | -0.173 | -0.167 | -0.160 | | -0.153 | -0.146 | -0.140 | | -0.133 | -0.126 |
| 0.6 | -0.207 | -0.207 | | -0.208 | -0.208 | -0.208 | | -0.208 | -0.209 | -0.209 | | -0.209 | -0.209 | -0.209 | | -0.210 | -0.210 |
| 0.8 | -0.207 | -0.214 | | -0.222 | -0.229 | -0.236 | | -0.243 | -0.250 | -0.258 | | -0.265 | -0.272 | -0.279 | | -0.286 | -0.294 |
| 1.0 | -0.207 | -0.221 | | -0.236 | -0.250 | -0.264 | | -0.278 | -0.292 | -0.306 | | -0.321 | -0.335 | -0.349 | | -0.363 | -0.377 |
| C2 | | | C3 | | | | C4 | | | | C5 | | | | C6 | | |

b) Second choice

|  | 1 | 0.8 | | 0.6 | 0.4 | 0.2 | | 0 | -0.2 | -0.4 | | -0.6 | -0.8 | -1 | | -1.2 | -1.4 |
| --- | --- | --- | --- | --- | --- | --- | --- | --- | --- | --- | --- | --- | --- | --- | --- | --- | --- |
| -0.2 | -0.187 | -0.166 | | -0.145 | -0.124 | -0.096 | | -0.069 | -0.041 | -0.013 | | 0.009 | 0.028 | 0.034 | | 0.043 | 0.064 |
| 0.0 | -0.187 | -0.168 | | -0.150 | -0.131 | -0.112 | | -0.094 | -0.075 | -0.056 | | -0.037 | -0.019 |  | | 0.005 | 0.011 |
| 0.2 | -0.187 | -0.171 | | -0.154 | -0.137 | -0.121 | | -0.104 | -0.088 | -0.076 | | -0.066 | -0.056 | -0.051 | | -0.051 | -0.042 |
| 0.4 | -0.187 | -0.173 | | -0.158 | -0.144 | -0.130 | | -0.118 | -0.113 | -0.108 | | -0.103 | -0.099 | -0.102 | | -0.106 | -0.111 |
| 0.6 | -0.187 | -0.175 | | -0.163 | -0.151 | -0.142 | | -0.142 | -0.141 | -0.141 | | -0.141 | -0.142 | -0.152 | | -0.162 | -0.172 |
| 0.8 | -0.187 | -0.177 | | -0.167 | -0.157 | -0.161 | | -0.165 | -0.169 | -0.174 | | -0.178 | -0.188 | -0.203 | | -0.218 | -0.233 |
| 1.0 | -0.187 | -0.179 | | -0.171 | -0.170 | -0.179 | | -0.188 | -0.198 | -0.207 | | -0.216 | -0.234 | -0.254 | | -0.274 | -0.294 |
| C2 | | | C3 | | | | C4 | | | | C5 | | | | C6 | | |

**Table S16**. The predicted most and second most reactive carbon atom of the condensed R-GPRI, , for a given and using the Hirshfeld population scheme to compute the atomic charge, , and the atomic radical Fukui function, , with the contribution of the bonded hydrogen atom summed in, for the reaction: **8** + ⸱CF_3_. represents the five likely reactive carbons in **8**, carbons labelled as C2-C6, see Scheme 2. Experimentally, the major products of this reaction are located at C6 and C4 with experimental yield ratio of 1.5:1. The kinetically most reactive atoms as computed using the enthalpy of activation barriers, Δ, are C3⪅C6≲C4 (and C3≲C4) with Δ of 25.89 kJ/mol, 26.23 kJ/mol, and 29.82 kJ/mol, respectively.

a) First choice

|  | 1 | 0.8 | | 0.6 | 0.4 | 0.2 | | 0 | -0.2 | -0.4 | | -0.6 | -0.8 | -1 | | -1.2 | -1.4 |
| --- | --- | --- | --- | --- | --- | --- | --- | --- | --- | --- | --- | --- | --- | --- | --- | --- | --- |
| -1 | -0.011 | -0.023 | | -0.040 | -0.065 | -0.090 | | -0.122 | -0.157 | -0.192 | | -0.227 | -0.262 | -0.297 | | -0.332 | -0.367 |
| -0.8 | -0.011 | -0.020 | | -0.030 | -0.050 | -0.070 | | -0.092 | -0.121 | -0.150 | | -0.180 | -0.209 | -0.238 | | -0.267 | -0.296 |
| -0.6 | -0.011 | -0.018 | | -0.025 | -0.036 | -0.051 | | -0.067 | -0.086 | -0.109 | | -0.132 | -0.155 | -0.178 | | -0.202 | -0.225 |
| -0.4 | -0.011 | -0.015 | | -0.019 | -0.023 | -0.032 | | -0.043 | -0.053 | -0.067 | | -0.084 | -0.102 | -0.119 | | -0.136 | -0.153 |
| -0.2 | -0.011 | -0.012 | | -0.014 | -0.016 | -0.017 | | -0.019 | -0.025 | -0.030 | | -0.037 | -0.048 | -0.059 | | -0.071 | -0.087 |
| 0.0 | -0.011 | -0.010 | | -0.009 | -0.008 | -0.007 | | -0.005 | -0.004 | -0.003 | | -0.002 | -0.001 |  | | -0.013 | -0.026 |
| 0.2 | -0.011 | -0.007 | | -0.003 | 0.000 | 0.004 | | 0.008 | 0.011 | 0.015 | | 0.019 | 0.023 | 0.026 | | 0.022 | 0.012 |
| C2 | | | C3 | | | | C4 | | | | C5 | | | | C6 | | |

b) Second choice

|  | 1 | 0.8 | | 0.6 | 0.4 | 0.2 | | 0 | -0.2 | -0.4 | | -0.6 | -0.8 | -1 | | -1.2 | -1.4 |
| --- | --- | --- | --- | --- | --- | --- | --- | --- | --- | --- | --- | --- | --- | --- | --- | --- | --- |
| -1 | 0.010 | -0.015 | | -0.035 | -0.052 | -0.087 | | -0.114 | -0.139 | -0.164 | | -0.189 | -0.220 | -0.259 | | -0.297 | -0.335 |
| -0.8 | 0.010 | -0.010 | | -0.030 | -0.039 | -0.063 | | -0.091 | -0.111 | -0.131 | | -0.151 | -0.174 | -0.207 | | -0.240 | -0.273 |
| -0.6 | 0.010 | -0.005 | | -0.021 | -0.031 | -0.039 | | -0.062 | -0.082 | -0.097 | | -0.113 | -0.128 | -0.155 | | -0.183 | -0.211 |
| -0.4 | 0.010 | -0.001 | | -0.011 | -0.022 | -0.028 | | -0.033 | -0.050 | -0.064 | | -0.074 | -0.085 | -0.103 | | -0.126 | -0.149 |
| -0.2 | 0.010 | 0.004 | | -0.002 | -0.007 | -0.013 | | -0.019 | -0.020 | -0.026 | | -0.036 | -0.042 | -0.052 | | -0.069 | -0.082 |
| 0.0 | 0.010 | 0.009 | | 0.008 | 0.007 | 0.006 | | 0.005 | 0.004 | 0.003 | | 0.002 | 0.001 |  | | -0.013 | -0.025 |
| 0.2 | 0.010 | 0.014 | | 0.018 | 0.021 | 0.025 | | 0.029 | 0.033 | 0.036 | | 0.040 | 0.042 | 0.032 | | 0.030 | 0.034 |
| C2 | | | C3 | | | | C4 | | | | C5 | | | | C6 | | |

**Table S17**. The predicted most and second most reactive carbon atom of the condensed R-GPRI, , for a given and using the Hirshfeld population scheme to compute the atomic charge, , and the atomic radical Fukui function, , with the contribution of the bonded hydrogen atom summed in, for the reaction: **8** + ⸱*i*-Pr. represents the five likely reactive carbons in **8**, carbons labelled as C2-C6, see Scheme 2. No products were isolated experimentally. The kinetically most reactive atoms as computed using the enthalpy of activation barriers, Δ, are C6≲C3 and C4 with Δ of 51.22 kJ/mol, 53.23 kJ/mol and 58.18 kJ/mol, respectively.

a) First choice

|  | 1 | 0.8 | | 0.6 | 0.4 | 0.2 | | 0 | -0.2 | -0.4 | | -0.6 | -0.8 | -1 | | -1.2 | -1.4 |
| --- | --- | --- | --- | --- | --- | --- | --- | --- | --- | --- | --- | --- | --- | --- | --- | --- | --- |
| -0.2 | -0.131 | -0.115 | | -0.098 | -0.082 | -0.066 | | -0.050 | -0.033 | -0.017 | | -0.001 | 0.016 | 0.026 | | 0.028 | 0.029 |
| 0.0 | -0.131 | -0.118 | | -0.105 | -0.092 | -0.079 | | -0.066 | -0.052 | -0.039 | | -0.026 | -0.013 |  | | -0.001 | -0.002 |
| 0.2 | -0.131 | -0.121 | | -0.111 | -0.103 | -0.096 | | -0.089 | -0.081 | -0.074 | | -0.066 | -0.059 | -0.059 | | -0.060 | -0.061 |
| 0.4 | -0.131 | -0.124 | | -0.121 | -0.119 | -0.117 | | -0.114 | -0.112 | -0.110 | | -0.108 | -0.112 | -0.119 | | -0.125 | -0.132 |
| 0.6 | -0.131 | -0.128 | | -0.131 | -0.134 | -0.137 | | -0.140 | -0.143 | -0.146 | | -0.153 | -0.166 | -0.178 | | -0.191 | -0.203 |
| 0.8 | -0.131 | -0.134 | | -0.142 | -0.150 | -0.158 | | -0.166 | -0.174 | -0.183 | | -0.201 | -0.219 | -0.238 | | -0.256 | -0.275 |
| 1.0 | -0.131 | -0.139 | | -0.152 | -0.165 | -0.179 | | -0.192 | -0.205 | -0.224 | | -0.249 | -0.273 | -0.297 | | -0.322 | -0.346 |
| C2 | | | C3 | | | | C4 | | | | C5 | | | | C6 | | |

b) Second choice

|  | 1 | 0.8 | | 0.6 | 0.4 | 0.2 | | 0 | -0.2 | -0.4 | | -0.6 | -0.8 | -1 | | -1.2 | -1.4 |
| --- | --- | --- | --- | --- | --- | --- | --- | --- | --- | --- | --- | --- | --- | --- | --- | --- | --- |
| -0.2 | -0.125 | -0.108 | | -0.090 | -0.072 | -0.055 | | -0.037 | -0.019 | -0.001 | | 0.016 | 0.025 | 0.032 | | 0.048 | 0.059 |
| 0.0 | -0.125 | -0.113 | | -0.100 | -0.088 | -0.075 | | -0.063 | -0.050 | -0.038 | | -0.025 | -0.013 |  | | 0.001 | 0.002 |
| 0.2 | -0.125 | -0.118 | | -0.111 | -0.101 | -0.091 | | -0.081 | -0.072 | -0.062 | | -0.058 | -0.059 | -0.052 | | -0.052 | -0.055 |
| 0.4 | -0.125 | -0.123 | | -0.118 | -0.111 | -0.104 | | -0.097 | -0.093 | -0.099 | | -0.106 | -0.106 | -0.103 | | -0.104 | -0.113 |
| 0.6 | -0.125 | -0.127 | | -0.124 | -0.120 | -0.117 | | -0.116 | -0.128 | -0.141 | | -0.149 | -0.152 | -0.155 | | -0.158 | -0.170 |
| 0.8 | -0.125 | -0.131 | | -0.130 | -0.130 | -0.130 | | -0.146 | -0.164 | -0.182 | | -0.191 | -0.199 | -0.207 | | -0.215 | -0.227 |
| 1.0 | -0.125 | -0.134 | | -0.137 | -0.139 | -0.151 | | -0.175 | -0.200 | -0.219 | | -0.232 | -0.245 | -0.259 | | -0.272 | -0.285 |
| C2 | | | C3 | | | | C4 | | | | C5 | | | | C6 | | |

**Table S18**. The predicted most and second most reactive carbon atom of the condensed R-GPRI, , for a given and using the Hirshfeld population scheme to compute the atomic charge, , and the atomic radical Fukui function, , with the contribution of the bonded hydrogen atom summed in, for the reaction: **9** + ⸱CF_3_. represents the five likely reactive carbons in **9**, carbons labelled as C2-C6, see Scheme 2. Experimentally, the major products of this reaction are located at C6 and C4 with experimental yield ratio of 1.1:1. The kinetically most reactive atoms as computed using the enthalpy of activation barriers, Δ, are C6, and C4⪅C5 with Δ of 23.57 kJ/mol, 27.87 kJ/mol, and 27.88 kJ/mol, respectively.

a) First choice

|  | 1 | 0.8 | | 0.6 | 0.4 | 0.2 | | 0 | -0.2 | -0.4 | | -0.6 | -0.8 | -1 | | -1.2 | -1.4 |
| --- | --- | --- | --- | --- | --- | --- | --- | --- | --- | --- | --- | --- | --- | --- | --- | --- | --- |
| -1 | -0.049 | -0.061 | | -0.073 | -0.085 | -0.109 | | -0.137 | -0.165 | -0.196 | | -0.246 | -0.296 | -0.347 | | -0.397 | -0.447 |
| -0.8 | -0.049 | -0.058 | | -0.066 | -0.075 | -0.087 | | -0.109 | -0.132 | -0.155 | | -0.191 | -0.234 | -0.277 | | -0.320 | -0.364 |
| -0.6 | -0.049 | -0.054 | | -0.060 | -0.065 | -0.070 | | -0.081 | -0.099 | -0.116 | | -0.135 | -0.171 | -0.208 | | -0.244 | -0.281 |
| -0.4 | -0.049 | -0.051 | | -0.053 | -0.055 | -0.057 | | -0.058 | -0.065 | -0.077 | | -0.088 | -0.109 | -0.138 | | -0.168 | -0.197 |
| -0.2 | -0.049 | -0.047 | | -0.046 | -0.045 | -0.043 | | -0.041 | -0.040 | -0.038 | | -0.043 | -0.049 | -0.069 | | -0.092 | -0.114 |
| 0.0 | -0.049 | -0.044 | | -0.039 | -0.034 | -0.029 | | -0.024 | -0.019 | -0.014 | | -0.009 | -0.004 |  | | -0.016 | -0.033 |
| 0.2 | -0.049 | -0.041 | | -0.033 | -0.024 | -0.016 | | -0.007 | 0.001 | 0.009 | | 0.017 | 0.025 | 0.027 | | 0.014 | 0.000 |
| C2 | | | C3 | | | | C4 | | | | C5 | | | | C6 | | |

b) Second choice

|  | 1 | 0.8 | | 0.6 | 0.4 | 0.2 | | 0 | -0.2 | -0.4 | | -0.6 | -0.8 | -1 | | -1.2 | -1.4 |
| --- | --- | --- | --- | --- | --- | --- | --- | --- | --- | --- | --- | --- | --- | --- | --- | --- | --- |
| -1 | 0.003 | -0.024 | | -0.053 | -0.081 | -0.098 | | -0.110 | -0.146 | -0.194 | | -0.222 | -0.250 | -0.278 | | -0.307 | -0.335 |
| -0.8 | 0.003 | -0.019 | | -0.041 | -0.064 | -0.084 | | -0.093 | -0.104 | -0.148 | | -0.177 | -0.200 | -0.222 | | -0.245 | -0.268 |
| -0.6 | 0.003 | -0.013 | | -0.030 | -0.047 | -0.065 | | -0.075 | -0.081 | -0.099 | | -0.133 | -0.150 | -0.167 | | -0.184 | -0.201 |
| -0.4 | 0.003 | -0.008 | | -0.019 | -0.031 | -0.042 | | -0.054 | -0.060 | -0.062 | | -0.080 | -0.099 | -0.111 | | -0.122 | -0.134 |
| -0.2 | 0.003 | -0.003 | | -0.008 | -0.014 | -0.020 | | -0.026 | -0.032 | -0.038 | | -0.037 | -0.047 | -0.055 | | -0.061 | -0.067 |
| 0.0 | 0.003 | 0.003 | | 0.003 | 0.002 | 0.002 | | 0.001 | 0.001 | 0.001 | | 0.001 | 0.000 |  | | -0.015 | -0.030 |
| 0.2 | 0.003 | 0.008 | | 0.013 | 0.019 | 0.024 | | 0.029 | 0.034 | 0.039 | | 0.045 | 0.041 | 0.034 | | 0.040 | 0.038 |
| C2 | | | C3 | | | | C4 | | | | C5 | | | | C6 | | |

**Table S19**. The predicted most and second most reactive carbon atom of the condensed R-GPRI, , for a given and using the Hirshfeld population scheme to compute the atomic charge, , and the atomic radical Fukui function, , with the contribution of the bonded hydrogen atom summed in, for the reaction: **9** + ⸱*i*-Pr. represents the five likely reactive carbons in **9**, carbons labelled as C2-C6, see Scheme 2. Experimentally, the major products of this reaction are located at C6 and C4 with experimental yield ratio of 10:1. The kinetically most reactive atoms as computed using the enthalpy of activation barriers, Δ, are C6, C4 and C5 with Δ of 43.22 kJ/mol, 54.31 kJ/mol and 58.38 kJ/mol, respectively.

a) First choice

|  | 1 | 0.8 | | 0.6 | 0.4 | 0.2 | | 0 | -0.2 | -0.4 | | -0.6 | -0.8 | -1 | | -1.2 | -1.4 |
| --- | --- | --- | --- | --- | --- | --- | --- | --- | --- | --- | --- | --- | --- | --- | --- | --- | --- |
| -0.2 | -0.164 | -0.145 | | -0.126 | -0.107 | -0.087 | | -0.068 | -0.049 | -0.030 | | -0.011 | 0.008 | 0.027 | | 0.033 | 0.031 |
| 0.0 | -0.164 | -0.148 | | -0.131 | -0.115 | -0.098 | | -0.082 | -0.065 | -0.049 | | -0.032 | -0.016 |  | | -0.005 | -0.009 |
| 0.2 | -0.164 | -0.151 | | -0.137 | -0.128 | -0.120 | | -0.111 | -0.103 | -0.094 | | -0.086 | -0.077 | -0.069 | | -0.061 | -0.066 |
| 0.4 | -0.164 | -0.153 | | -0.150 | -0.149 | -0.147 | | -0.146 | -0.144 | -0.143 | | -0.141 | -0.140 | -0.138 | | -0.137 | -0.136 |
| 0.6 | -0.164 | -0.159 | | -0.164 | -0.170 | -0.175 | | -0.181 | -0.186 | -0.191 | | -0.197 | -0.202 | -0.208 | | -0.213 | -0.219 |
| 0.8 | -0.164 | -0.166 | | -0.178 | -0.191 | -0.203 | | -0.215 | -0.228 | -0.240 | | -0.252 | -0.265 | -0.277 | | -0.289 | -0.302 |
| 1.0 | -0.164 | -0.173 | | -0.192 | -0.211 | -0.231 | | -0.250 | -0.269 | -0.289 | | -0.308 | -0.327 | -0.347 | | -0.366 | -0.385 |
| C2 | | | C3 | | | | C4 | | | | C5 | | | | C6 | | |

b) Second choice

|  | 1 | 0.8 | | 0.6 | 0.4 | 0.2 | | 0 | -0.2 | -0.4 | | -0.6 | -0.8 | -1 | | -1.2 | -1.4 |
| --- | --- | --- | --- | --- | --- | --- | --- | --- | --- | --- | --- | --- | --- | --- | --- | --- | --- |
| -0.2 | -0.154 | -0.131 | | -0.109 | -0.087 | -0.064 | | -0.042 | -0.019 | 0.002 | | 0.021 | 0.031 | 0.034 | | 0.046 | 0.063 |
| 0.0 | -0.154 | -0.138 | | -0.123 | -0.107 | -0.092 | | -0.077 | -0.061 | -0.046 | | -0.030 | -0.015 |  | | 0.000 | 0.001 |
| 0.2 | -0.154 | -0.145 | | -0.137 | -0.123 | -0.109 | | -0.096 | -0.082 | -0.068 | | -0.054 | -0.050 | -0.055 | | -0.061 | -0.052 |
| 0.4 | -0.154 | -0.152 | | -0.142 | -0.131 | -0.120 | | -0.109 | -0.098 | -0.087 | | -0.089 | -0.100 | -0.111 | | -0.122 | -0.133 |
| 0.6 | -0.154 | -0.156 | | -0.148 | -0.139 | -0.131 | | -0.123 | -0.115 | -0.118 | | -0.134 | -0.150 | -0.167 | | -0.184 | -0.200 |
| 0.8 | -0.154 | -0.159 | | -0.153 | -0.148 | -0.142 | | -0.137 | -0.135 | -0.157 | | -0.179 | -0.200 | -0.222 | | -0.245 | -0.267 |
| 1.0 | -0.154 | -0.161 | | -0.159 | -0.156 | -0.153 | | -0.150 | -0.168 | -0.196 | | -0.223 | -0.251 | -0.278 | | -0.306 | -0.334 |
| C2 | | | C3 | | | | C4 | | | | C5 | | | | C6 | | |

**Table S20**. The predicted most and second most reactive carbon atom of the condensed R-GPRI, , for a given and using the Hirshfeld population scheme to compute the atomic charge, , and the atomic radical Fukui function, , with the contribution of the bonded hydrogen atom summed in, for the reaction: **10** + ⸱CF_3_. represents the five likely reactive carbons in **10**, carbons labelled as C2-C6, see Scheme 2. Experimentally, the major products of this reaction are located at C2 and C6 with experimental yield ratio of 1.6:1. The kinetically most reactive atoms as computed using the enthalpy of activation barriers, Δ, are C6⪅C2⪅C5 (and C6⪅C5) with Δ of 22.93 kJ/mol, 23.40 kJ/mol, and 24.60 kJ/mol, respectively.

a) First choice

|  | 1 | 0.8 | | 0.6 | 0.4 | 0.2 | | 0 | -0.2 | -0.4 | | -0.6 | -0.8 | -1 | | -1.2 | -1.4 |
| --- | --- | --- | --- | --- | --- | --- | --- | --- | --- | --- | --- | --- | --- | --- | --- | --- | --- |
| -1 | -0.023 | -0.046 | | -0.069 | -0.091 | -0.114 | | -0.137 | -0.160 | -0.182 | | -0.205 | -0.231 | -0.268 | | -0.306 | -0.343 |
| -0.8 | -0.023 | -0.041 | | -0.059 | -0.076 | -0.094 | | -0.112 | -0.129 | -0.147 | | -0.165 | -0.183 | -0.215 | | -0.247 | -0.279 |
| -0.6 | -0.023 | -0.036 | | -0.049 | -0.061 | -0.074 | | -0.087 | -0.099 | -0.112 | | -0.125 | -0.137 | -0.161 | | -0.188 | -0.214 |
| -0.4 | -0.023 | -0.031 | | -0.039 | -0.046 | -0.054 | | -0.062 | -0.069 | -0.077 | | -0.085 | -0.092 | -0.107 | | -0.129 | -0.149 |
| -0.2 | -0.023 | -0.026 | | -0.028 | -0.031 | -0.034 | | -0.036 | -0.039 | -0.042 | | -0.044 | -0.047 | -0.053 | | -0.069 | -0.085 |
| 0.0 | -0.023 | -0.021 | | -0.018 | -0.016 | -0.014 | | -0.011 | -0.009 | -0.007 | | -0.004 | -0.002 |  | | -0.010 | -0.021 |
| 0.2 | -0.023 | -0.016 | | -0.008 | -0.001 | 0.005 | | 0.010 | 0.014 | 0.017 | | 0.017 | 0.015 | 0.013 | | 0.011 | 0.009 |
| C2 | | | C3 | | | | C4 | | | | C5 | | | | C6 | | |

b) Second choice

|  | 1 | 0.8 | | 0.6 | 0.4 | 0.2 | | 0 | -0.2 | -0.4 | | -0.6 | -0.8 | -1 | | -1.2 | -1.4 |
| --- | --- | --- | --- | --- | --- | --- | --- | --- | --- | --- | --- | --- | --- | --- | --- | --- | --- |
| -1 | -0.007 | -0.020 | | -0.034 | -0.047 | -0.060 | | -0.082 | -0.119 | -0.156 | | -0.194 | -0.228 | -0.251 | | -0.274 | -0.296 |
| -0.8 | -0.007 | -0.017 | | -0.028 | -0.038 | -0.049 | | -0.060 | -0.087 | -0.119 | | -0.151 | -0.183 | -0.200 | | -0.219 | -0.240 |
| -0.6 | -0.007 | -0.014 | | -0.022 | -0.030 | -0.038 | | -0.046 | -0.055 | -0.081 | | -0.108 | -0.134 | -0.150 | | -0.163 | -0.185 |
| -0.4 | -0.007 | -0.012 | | -0.017 | -0.022 | -0.026 | | -0.031 | -0.036 | -0.043 | | -0.065 | -0.086 | -0.100 | | -0.111 | -0.129 |
| -0.2 | -0.007 | -0.009 | | -0.011 | -0.013 | -0.015 | | -0.017 | -0.019 | -0.021 | | -0.024 | -0.037 | -0.050 | | -0.060 | -0.074 |
| 0.0 | -0.007 | -0.006 | | -0.005 | -0.005 | -0.004 | | -0.003 | -0.002 | -0.002 | | -0.001 | -0.001 |  | | -0.009 | -0.018 |
| 0.2 | -0.007 | -0.003 | | 0.000 | 0.003 | 0.006 | | 0.013 | 0.020 | 0.019 | | 0.021 | 0.024 | 0.028 | | 0.032 | 0.035 |
| C2 | | | C3 | | | | C4 | | | | C5 | | | | C6 | | |

**Table S21**. The predicted most and second most reactive carbon atom of the condensed R-GPRI, , for a given and using the Hirshfeld population scheme to compute the atomic charge, , and the atomic radical Fukui function, , with the contribution of the bonded hydrogen atom summed in, for the reaction: **10** + ⸱*i*-Pr. represents the five likely reactive carbons in **10**, carbons labelled as C2-C6, see Scheme 2. No products were isolated experimentally. The kinetically most reactive atoms as computed using the enthalpy of activation barriers, Δ, are C6⪅C2 with Δ of 54.28 kJ/mol and 54.39 kJ/mol, respectively.

a) First choice

|  | 1 | 0.8 | | 0.6 | 0.4 | 0.2 | | 0 | -0.2 | -0.4 | | -0.6 | -0.8 | -1 | | -1.2 | -1.4 |
| --- | --- | --- | --- | --- | --- | --- | --- | --- | --- | --- | --- | --- | --- | --- | --- | --- | --- |
| -0.2 | -0.104 | -0.088 | | -0.072 | -0.056 | -0.041 | | -0.025 | -0.010 | -0.001 | | 0.003 | 0.008 | 0.013 | | 0.018 | 0.022 |
| 0.0 | -0.104 | -0.093 | | -0.083 | -0.073 | -0.062 | | -0.052 | -0.041 | -0.031 | | -0.020 | -0.010 |  | | -0.002 | -0.005 |
| 0.2 | -0.104 | -0.099 | | -0.094 | -0.089 | -0.084 | | -0.079 | -0.074 | -0.068 | | -0.063 | -0.058 | -0.053 | | -0.058 | -0.065 |
| 0.4 | -0.104 | -0.104 | | -0.105 | -0.105 | -0.105 | | -0.105 | -0.106 | -0.106 | | -0.106 | -0.107 | -0.107 | | -0.112 | -0.125 |
| 0.6 | -0.104 | -0.110 | | -0.115 | -0.121 | -0.127 | | -0.132 | -0.138 | -0.144 | | -0.149 | -0.155 | -0.161 | | -0.168 | -0.185 |
| 0.8 | -0.104 | -0.115 | | -0.126 | -0.137 | -0.148 | | -0.159 | -0.170 | -0.181 | | -0.192 | -0.204 | -0.215 | | -0.226 | -0.246 |
| 1.0 | -0.104 | -0.120 | | -0.137 | -0.153 | -0.170 | | -0.186 | -0.203 | -0.219 | | -0.235 | -0.252 | -0.268 | | -0.285 | -0.306 |
| C2 | | | C3 | | | | C4 | | | | C5 | | | | C6 | | |

b) Second choice

|  | 1 | 0.8 | | 0.6 | 0.4 | 0.2 | | 0 | -0.2 | -0.4 | | -0.6 | -0.8 | -1 | | -1.2 | -1.4 |
| --- | --- | --- | --- | --- | --- | --- | --- | --- | --- | --- | --- | --- | --- | --- | --- | --- | --- |
| -0.2 | -0.093 | -0.079 | | -0.065 | -0.051 | -0.037 | | -0.023 | -0.009 | 0.004 | | 0.018 | 0.026 | 0.028 | | 0.030 | 0.032 |
| 0.0 | -0.093 | -0.084 | | -0.074 | -0.065 | -0.056 | | -0.046 | -0.037 | -0.028 | | -0.018 | -0.009 |  | | -0.001 | -0.001 |
| 0.2 | -0.093 | -0.088 | | -0.084 | -0.079 | -0.074 | | -0.069 | -0.065 | -0.060 | | -0.055 | -0.050 | -0.050 | | -0.049 | -0.043 |
| 0.4 | -0.093 | -0.093 | | -0.093 | -0.093 | -0.093 | | -0.093 | -0.092 | -0.092 | | -0.092 | -0.092 | -0.100 | | -0.108 | -0.108 |
| 0.6 | -0.093 | -0.098 | | -0.103 | -0.107 | -0.111 | | -0.116 | -0.120 | -0.125 | | -0.129 | -0.134 | -0.150 | | -0.167 | -0.173 |
| 0.8 | -0.093 | -0.103 | | -0.111 | -0.120 | -0.130 | | -0.139 | -0.148 | -0.157 | | -0.166 | -0.178 | -0.200 | | -0.223 | -0.237 |
| 1.0 | -0.093 | -0.107 | | -0.121 | -0.134 | -0.148 | | -0.162 | -0.176 | -0.189 | | -0.203 | -0.223 | -0.251 | | -0.278 | -0.302 |
| C2 | | | C3 | | | | C4 | | | | C5 | | | | C6 | | |

**Table S22**. The predicted most and second most reactive carbon atom of the condensed R-GPRI, , for a given and using the Hirshfeld population scheme to compute the atomic charge, , and the atomic radical Fukui function, , with the contribution of the bonded hydrogen atom summed in, for the reaction: **11** + ⸱CF_3_. represents the five likely reactive carbons in **11**, carbons labelled as C2-C6, see Scheme 2. Experimentally, the major products of this reaction are located at C2 and C6 with experimental yield ratio of 1.4:1. The kinetically most reactive atoms as computed using the enthalpy of activation barriers, Δ, are C2⪅C6⪅C5 (and C2⪅C5) with Δ of 22.90 kJ/mol, 23.15 kJ/mol, and 23.63 kJ/mol, respectively.

a) First choice

|  | 1 | 0.8 | | 0.6 | 0.4 | 0.2 | | 0 | -0.2 | -0.4 | | -0.6 | -0.8 | -1 | | -1.2 | -1.4 |
| --- | --- | --- | --- | --- | --- | --- | --- | --- | --- | --- | --- | --- | --- | --- | --- | --- | --- |
| -1 | -0.029 | -0.039 | | -0.049 | -0.059 | -0.068 | | -0.095 | -0.127 | -0.166 | | -0.204 | -0.243 | -0.281 | | -0.320 | -0.358 |
| -0.8 | -0.029 | -0.036 | | -0.044 | -0.051 | -0.058 | | -0.070 | -0.096 | -0.126 | | -0.159 | -0.192 | -0.225 | | -0.258 | -0.291 |
| -0.6 | -0.029 | -0.034 | | -0.039 | -0.043 | -0.048 | | -0.053 | -0.066 | -0.087 | | -0.114 | -0.141 | -0.168 | | -0.196 | -0.223 |
| -0.4 | -0.029 | -0.031 | | -0.033 | -0.036 | -0.038 | | -0.040 | -0.042 | -0.052 | | -0.069 | -0.091 | -0.112 | | -0.134 | -0.156 |
| -0.2 | -0.029 | -0.029 | | -0.028 | -0.028 | -0.028 | | -0.028 | -0.027 | -0.026 | | -0.028 | -0.040 | -0.056 | | -0.072 | -0.088 |
| 0.0 | -0.029 | -0.026 | | -0.023 | -0.020 | -0.018 | | -0.015 | -0.011 | -0.008 | | -0.005 | -0.002 |  | | -0.015 | -0.031 |
| 0.2 | -0.029 | -0.024 | | -0.018 | -0.013 | -0.008 | | -0.002 | 0.003 | 0.008 | | 0.014 | 0.020 | 0.024 | | 0.011 | -0.002 |
| C2 | | | C3 | | | | C4 | | | | C5 | | | | C6 | | |

b) Second choice

|  | 1 | 0.8 | | 0.6 | 0.4 | 0.2 | | 0 | -0.2 | -0.4 | | -0.6 | -0.8 | -1 | | -1.2 | -1.4 |
| --- | --- | --- | --- | --- | --- | --- | --- | --- | --- | --- | --- | --- | --- | --- | --- | --- | --- |
| -1 | 0.043 | 0.018 | | -0.007 | -0.032 | -0.063 | | -0.089 | -0.126 | -0.158 | | -0.190 | -0.221 | -0.253 | | -0.285 | -0.317 |
| -0.8 | 0.043 | 0.022 | | 0.001 | -0.019 | -0.043 | | -0.065 | -0.094 | -0.123 | | -0.149 | -0.176 | -0.202 | | -0.229 | -0.256 |
| -0.6 | 0.043 | 0.026 | | 0.010 | -0.007 | -0.024 | | -0.044 | -0.060 | -0.087 | | -0.109 | -0.130 | -0.152 | | -0.173 | -0.195 |
| -0.4 | 0.043 | 0.031 | | 0.018 | 0.005 | -0.007 | | -0.020 | -0.035 | -0.048 | | -0.068 | -0.084 | -0.101 | | -0.118 | -0.134 |
| -0.2 | 0.043 | 0.035 | | 0.027 | 0.018 | 0.009 | | 0.001 | -0.007 | -0.016 | | -0.026 | -0.039 | -0.050 | | -0.062 | -0.073 |
| 0.0 | 0.043 | 0.039 | | 0.035 | 0.030 | 0.026 | | 0.021 | 0.017 | 0.013 | | 0.008 | 0.004 |  | | -0.010 | -0.020 |
| 0.2 | 0.043 | 0.044 | | 0.043 | 0.043 | 0.043 | | 0.043 | 0.042 | 0.042 | | 0.042 | 0.037 | 0.025 | | 0.031 | 0.036 |
| C2 | | | C3 | | | | C4 | | | | C5 | | | | C6 | | |

**Table S23**. The predicted most and second most reactive carbon atom of the condensed R-GPRI, , for a given and using the Hirshfeld population scheme to compute the atomic charge, , and the atomic radical Fukui function, , with the contribution of the bonded hydrogen atom summed in, for the reaction: **11** + ⸱*i*-Pr. represents the five likely reactive carbons in **11**, carbons labelled as C2-C6, see Scheme 2. Experimentally, the major product of this reaction is located at C2. No second product was isolated experimentally. The kinetically most reactive atoms as computed using the enthalpy of activation barriers, Δ, are C5 and C2≲C6 with Δ of 43.57 kJ/mol, 48.94 kJ/mol, and 51.45 kJ/mol, respectively.

a) First choice

|  | 1 | 0.8 | | 0.6 | 0.4 | 0.2 | | 0 | -0.2 | -0.4 | | -0.6 | -0.8 | -1 | | -1.2 | -1.4 |
| --- | --- | --- | --- | --- | --- | --- | --- | --- | --- | --- | --- | --- | --- | --- | --- | --- | --- |
| -0.2 | -0.154 | -0.136 | | -0.118 | -0.100 | -0.082 | | -0.065 | -0.047 | -0.029 | | -0.011 | 0.006 | 0.024 | | 0.025 | 0.025 |
| 0.0 | -0.154 | -0.139 | | -0.123 | -0.108 | -0.092 | | -0.077 | -0.061 | -0.046 | | -0.030 | -0.015 |  | | -0.003 | -0.006 |
| 0.2 | -0.154 | -0.141 | | -0.128 | -0.115 | -0.102 | | -0.089 | -0.076 | -0.070 | | -0.065 | -0.060 | -0.056 | | -0.052 | -0.048 |
| 0.4 | -0.154 | -0.143 | | -0.133 | -0.122 | -0.112 | | -0.107 | -0.108 | -0.109 | | -0.110 | -0.111 | -0.112 | | -0.113 | -0.115 |
| 0.6 | -0.154 | -0.146 | | -0.138 | -0.130 | -0.129 | | -0.135 | -0.142 | -0.148 | | -0.155 | -0.162 | -0.168 | | -0.176 | -0.182 |
| 0.8 | -0.154 | -0.148 | | -0.143 | -0.139 | -0.151 | | -0.163 | -0.176 | -0.188 | | -0.200 | -0.212 | -0.225 | | -0.237 | -0.249 |
| 1.0 | -0.154 | -0.151 | | -0.148 | -0.156 | -0.174 | | -0.192 | -0.209 | -0.227 | | -0.245 | -0.263 | -0.281 | | -0.299 | -0.317 |
| C2 | | | C3 | | | | C4 | | | | C5 | | | | C6 | | |

b) Second choice

|  | 1 | 0.8 | | 0.6 | 0.4 | 0.2 | | 0 | -0.2 | -0.4 | | -0.6 | -0.8 | -1 | | -1.2 | -1.4 |
| --- | --- | --- | --- | --- | --- | --- | --- | --- | --- | --- | --- | --- | --- | --- | --- | --- | --- |
| -0.2 | -0.102 | -0.086 | | -0.070 | -0.054 | -0.038 | | -0.023 | -0.007 | 0.008 | | 0.024 | 0.025 | 0.025 | | 0.042 | 0.059 |
| 0.0 | -0.102 | -0.092 | | -0.082 | -0.071 | -0.061 | | -0.051 | -0.040 | -0.030 | | -0.020 | -0.010 |  | | 0.004 | 0.009 |
| 0.2 | -0.102 | -0.098 | | -0.093 | -0.088 | -0.083 | | -0.079 | -0.074 | -0.063 | | -0.053 | -0.051 | -0.050 | | -0.049 | -0.047 |
| 0.4 | -0.102 | -0.103 | | -0.104 | -0.105 | -0.106 | | -0.101 | -0.091 | -0.089 | | -0.093 | -0.097 | -0.101 | | -0.105 | -0.109 |
| 0.6 | -0.102 | -0.109 | | -0.115 | -0.122 | -0.122 | | -0.113 | -0.116 | -0.125 | | -0.134 | -0.143 | -0.152 | | -0.161 | -0.169 |
| 0.8 | -0.102 | -0.114 | | -0.127 | -0.137 | -0.131 | | -0.133 | -0.146 | -0.160 | | -0.174 | -0.188 | -0.202 | | -0.217 | -0.230 |
| 1.0 | -0.102 | -0.120 | | -0.138 | -0.144 | -0.141 | | -0.158 | -0.177 | -0.196 | | -0.215 | -0.234 | -0.253 | | -0.272 | -0.291 |
| C2 | | | C3 | | | | C4 | | | | C5 | | | | C6 | | |

**Table S24**. The predicted most and second most reactive carbon atom of the condensed R-GPRI, , for a given and using the Hirshfeld population scheme to compute the atomic charge, , and the atomic radical Fukui function, , with the contribution of the bonded hydrogen atom summed in, for the reaction: **12** + ⸱CF_3_. represents the five likely reactive carbons in **12**, carbons labelled as C2-C6, see Scheme 2. Experimentally, the major products of this reaction are located at C2, C6, and C4 with experimental yield ratio of 2.1:1.3:1. The kinetically most reactive atoms as computed using the enthalpy of activation barriers, Δ, are C2⪅C6⪅C4 (and C2⪅C4) with Δ of 22.97 kJ/mol, 23.77 kJ/mol, and 23.98 kJ/mol, respectively.

a) First choice

|  | 1 | 0.8 | | 0.6 | 0.4 | 0.2 | | 0 | -0.2 | -0.4 | | -0.6 | -0.8 | -1 | | -1.2 | -1.4 |
| --- | --- | --- | --- | --- | --- | --- | --- | --- | --- | --- | --- | --- | --- | --- | --- | --- | --- |
| -1 | 0.004 | -0.008 | | -0.031 | -0.061 | -0.092 | | -0.124 | -0.161 | -0.198 | | -0.236 | -0.273 | -0.310 | | -0.348 | -0.385 |
| -0.8 | 0.004 | -0.006 | | -0.020 | -0.045 | -0.070 | | -0.095 | -0.124 | -0.155 | | -0.186 | -0.217 | -0.248 | | -0.279 | -0.311 |
| -0.6 | 0.004 | -0.004 | | -0.011 | -0.029 | -0.048 | | -0.067 | -0.087 | -0.111 | | -0.136 | -0.161 | -0.186 | | -0.211 | -0.236 |
| -0.4 | 0.004 | -0.001 | | -0.006 | -0.012 | -0.026 | | -0.040 | -0.054 | -0.068 | | -0.087 | -0.105 | -0.124 | | -0.143 | -0.162 |
| -0.2 | 0.004 | 0.001 | | -0.002 | -0.004 | -0.007 | | -0.013 | -0.021 | -0.029 | | -0.038 | -0.050 | -0.062 | | -0.075 | -0.087 |
| 0.0 | 0.004 | 0.003 | | 0.003 | 0.003 | 0.002 | | 0.002 | 0.001 | 0.001 | | 0.001 | 0.000 |  | | -0.015 | -0.029 |
| 0.2 | 0.004 | 0.006 | | 0.007 | 0.009 | 0.011 | | 0.013 | 0.015 | 0.017 | | 0.019 | 0.021 | 0.023 | | 0.012 | 0.000 |
| C2 | | | C3 | | | | C4 | | | | C5 | | | | C6 | | |

b) Second choice

|  | 1 | 0.8 | | 0.6 | 0.4 | 0.2 | | 0 | -0.2 | -0.4 | | -0.6 | -0.8 | -1 | | -1.2 | -1.4 |
| --- | --- | --- | --- | --- | --- | --- | --- | --- | --- | --- | --- | --- | --- | --- | --- | --- | --- |
| -1 | 0.029 | -0.001 | | -0.020 | -0.049 | -0.086 | | -0.122 | -0.152 | -0.182 | | -0.213 | -0.243 | -0.274 | | -0.311 | -0.347 |
| -0.8 | 0.029 | 0.005 | | -0.015 | -0.030 | -0.061 | | -0.093 | -0.119 | -0.144 | | -0.169 | -0.194 | -0.219 | | -0.250 | -0.281 |
| -0.6 | 0.029 | 0.010 | | -0.009 | -0.018 | -0.037 | | -0.062 | -0.087 | -0.106 | | -0.125 | -0.145 | -0.164 | | -0.190 | -0.216 |
| -0.4 | 0.029 | 0.016 | | 0.002 | -0.011 | -0.016 | | -0.031 | -0.049 | -0.068 | | -0.082 | -0.095 | -0.110 | | -0.130 | -0.150 |
| -0.2 | 0.029 | 0.021 | | 0.013 | 0.004 | -0.004 | | -0.010 | -0.012 | -0.025 | | -0.037 | -0.046 | -0.055 | | -0.069 | -0.084 |
| 0.0 | 0.029 | 0.026 | | 0.024 | 0.021 | 0.018 | | 0.015 | 0.012 | 0.009 | | 0.006 | 0.003 |  | | -0.009 | -0.018 |
| 0.2 | 0.029 | 0.032 | | 0.034 | 0.037 | 0.039 | | 0.042 | 0.045 | 0.047 | | 0.049 | 0.036 | 0.024 | | 0.025 | 0.027 |
| C2 | | | C3 | | | | C4 | | | | C5 | | | | C6 | | |

**Table S25**. The predicted most and second most reactive carbon atom of the condensed R-GPRI, , for a given and using the Hirshfeld population scheme to compute the atomic charge, , and the atomic radical Fukui function, , with the contribution of the bonded hydrogen atom summed in, for the reaction: **12** + ⸱*i*-Pr. represents the five likely reactive carbons in **12**, carbons labelled as C2-C6, see Scheme 2. No products were isolated experimentally. The kinetically most reactive atoms as computed using the enthalpy of activation barriers, Δ, are C4⪅C6⪅C2 (and C4≲C2) with Δ of 45.42 kJ/mol, 47.02 kJ/mol, and 49.23 kJ/mol, respectively.

a) First choice

|  | 1 | 0.8 | | 0.6 | 0.4 | 0.2 | | 0 | -0.2 | -0.4 | | -0.6 | -0.8 | -1 | | -1.2 | -1.4 |
| --- | --- | --- | --- | --- | --- | --- | --- | --- | --- | --- | --- | --- | --- | --- | --- | --- | --- |
| -0.2 | -0.147 | -0.130 | | -0.112 | -0.095 | -0.078 | | -0.061 | -0.044 | -0.027 | | -0.010 | 0.007 | 0.023 | | 0.025 | 0.028 |
| 0.0 | -0.147 | -0.132 | | -0.117 | -0.103 | -0.088 | | -0.073 | -0.059 | -0.044 | | -0.029 | -0.015 |  | | 0.000 | 0.001 |
| 0.2 | -0.147 | -0.134 | | -0.122 | -0.110 | -0.098 | | -0.085 | -0.073 | -0.066 | | -0.062 | -0.062 | -0.062 | | -0.062 | -0.062 |
| 0.4 | -0.147 | -0.137 | | -0.127 | -0.117 | -0.107 | | -0.101 | -0.102 | -0.106 | | -0.112 | -0.118 | -0.124 | | -0.130 | -0.136 |
| 0.6 | -0.147 | -0.139 | | -0.132 | -0.124 | -0.121 | | -0.128 | -0.137 | -0.149 | | -0.162 | -0.174 | -0.186 | | -0.199 | -0.211 |
| 0.8 | -0.147 | -0.142 | | -0.137 | -0.132 | -0.143 | | -0.156 | -0.174 | -0.193 | | -0.211 | -0.230 | -0.248 | | -0.267 | -0.285 |
| 1.0 | -0.147 | -0.144 | | -0.142 | -0.146 | -0.165 | | -0.187 | -0.211 | -0.236 | | -0.261 | -0.286 | -0.310 | | -0.335 | -0.360 |
| C2 | | | C3 | | | | C4 | | | | C5 | | | | C6 | | |

b) Second choice

|  | 1 | 0.8 | | 0.6 | 0.4 | 0.2 | | 0 | -0.2 | -0.4 | | -0.6 | -0.8 | -1 | | -1.2 | -1.4 |
| --- | --- | --- | --- | --- | --- | --- | --- | --- | --- | --- | --- | --- | --- | --- | --- | --- | --- |
| -0.2 | -0.092 | -0.077 | | -0.062 | -0.048 | -0.033 | | -0.018 | -0.004 | 0.011 | | 0.017 | 0.020 | 0.024 | | 0.041 | 0.058 |
| 0.0 | -0.092 | -0.082 | | -0.073 | -0.064 | -0.055 | | -0.046 | -0.037 | -0.028 | | -0.018 | -0.009 |  | | 0.003 | 0.006 |
| 0.2 | -0.092 | -0.088 | | -0.084 | -0.081 | -0.077 | | -0.073 | -0.070 | -0.062 | | -0.062 | -0.059 | -0.055 | | -0.057 | -0.060 |
| 0.4 | -0.092 | -0.093 | | -0.095 | -0.097 | -0.099 | | -0.098 | -0.100 | -0.104 | | -0.106 | -0.108 | -0.110 | | -0.117 | -0.125 |
| 0.6 | -0.092 | -0.099 | | -0.106 | -0.114 | -0.117 | | -0.125 | -0.135 | -0.143 | | -0.150 | -0.157 | -0.164 | | -0.177 | -0.191 |
| 0.8 | -0.092 | -0.104 | | -0.117 | -0.130 | -0.137 | | -0.155 | -0.168 | -0.181 | | -0.194 | -0.207 | -0.219 | | -0.238 | -0.256 |
| 1.0 | -0.092 | -0.110 | | -0.128 | -0.139 | -0.162 | | -0.183 | -0.201 | -0.219 | | -0.238 | -0.256 | -0.274 | | -0.298 | -0.322 |
| C2 | | | C3 | | | | C4 | | | | C5 | | | | C6 | | |

**Table S26**. The predicted most and second most reactive carbon atom of the condensed R-GPRI, , for a given and using the Hirshfeld population scheme to compute the atomic charge, , and the atomic radical Fukui function, , with the contribution of the bonded hydrogen atom summed in, for the reaction: **13** + ⸱CF_3_. represents the five likely reactive carbons in **13**, carbons labelled as C2-C6, see Scheme 2. Experimentally, the major products of this reaction are located at C6 and C4 with experimental yield ratio of 4:1. The kinetically most reactive atoms as computed using the enthalpy of activation barriers, Δ, are C6⪅C5≲C4 (and C6<C4) with Δ of 24.98 kJ/mol, 26.85 kJ/mol, and 29.88 kJ/mol, respectively.

a) First choice

|  | 1 | 0.8 | | 0.6 | 0.4 | 0.2 | | 0 | -0.2 | -0.4 | | -0.6 | -0.8 | -1 | | -1.2 | -1.4 |
| --- | --- | --- | --- | --- | --- | --- | --- | --- | --- | --- | --- | --- | --- | --- | --- | --- | --- |
| -1 | 0.008 | -0.024 | | -0.061 | -0.099 | -0.137 | | -0.175 | -0.213 | -0.251 | | -0.289 | -0.327 | -0.364 | | -0.402 | -0.440 |
| -0.8 | 0.008 | -0.016 | | -0.047 | -0.077 | -0.108 | | -0.139 | -0.169 | -0.200 | | -0.230 | -0.261 | -0.292 | | -0.322 | -0.353 |
| -0.6 | 0.008 | -0.009 | | -0.032 | -0.056 | -0.079 | | -0.102 | -0.125 | -0.149 | | -0.172 | -0.195 | -0.219 | | -0.242 | -0.265 |
| -0.4 | 0.008 | -0.002 | | -0.018 | -0.034 | -0.050 | | -0.066 | -0.082 | -0.098 | | -0.114 | -0.130 | -0.146 | | -0.162 | -0.178 |
| -0.2 | 0.008 | 0.005 | | -0.003 | -0.012 | -0.021 | | -0.029 | -0.038 | -0.047 | | -0.055 | -0.064 | -0.073 | | -0.082 | -0.094 |
| 0.0 | 0.008 | 0.007 | | 0.006 | 0.005 | 0.005 | | 0.004 | 0.003 | 0.002 | | 0.002 | 0.001 |  | | -0.014 | -0.029 |
| 0.2 | 0.008 | 0.009 | | 0.011 | 0.012 | 0.014 | | 0.016 | 0.017 | 0.019 | | 0.020 | 0.022 | 0.020 | | 0.009 | -0.001 |
| C2 | | | C3 | | | | C4 | | | | C5 | | | | C6 | | |

b) Second choice

|  | 1 | 0.8 | | 0.6 | 0.4 | 0.2 | | 0 | -0.2 | -0.4 | | -0.6 | -0.8 | -1 | | -1.2 | -1.4 |
| --- | --- | --- | --- | --- | --- | --- | --- | --- | --- | --- | --- | --- | --- | --- | --- | --- | --- |
| -1 | 0.014 | -0.005 | | -0.017 | -0.053 | -0.090 | | -0.126 | -0.162 | -0.198 | | -0.234 | -0.271 | -0.307 | | -0.343 | -0.379 |
| -0.8 | 0.014 | -0.002 | | -0.013 | -0.035 | -0.065 | | -0.095 | -0.125 | -0.155 | | -0.185 | -0.215 | -0.245 | | -0.276 | -0.306 |
| -0.6 | 0.014 | 0.000 | | -0.008 | -0.017 | -0.040 | | -0.064 | -0.088 | -0.112 | | -0.136 | -0.160 | -0.184 | | -0.208 | -0.232 |
| -0.4 | 0.014 | 0.002 | | -0.003 | -0.009 | -0.016 | | -0.034 | -0.052 | -0.069 | | -0.087 | -0.105 | -0.123 | | -0.141 | -0.159 |
| -0.2 | 0.014 | 0.006 | | 0.002 | -0.002 | -0.005 | | -0.008 | -0.015 | -0.026 | | -0.038 | -0.050 | -0.061 | | -0.074 | -0.090 |
| 0.0 | 0.014 | 0.013 | | 0.011 | 0.010 | 0.009 | | 0.007 | 0.006 | 0.004 | | 0.003 | 0.001 |  | | -0.012 | -0.024 |
| 0.2 | 0.014 | 0.020 | | 0.026 | 0.032 | 0.038 | | 0.044 | 0.049 | 0.050 | | 0.040 | 0.030 | 0.023 | | 0.025 | 0.027 |
| C2 | | | C3 | | | | C4 | | | | C5 | | | | C6 | | |

**Table S27**. The predicted most and second most reactive carbon atom of the condensed R-GPRI, , for a given and using the Hirshfeld population scheme to compute the atomic charge, , and the atomic radical Fukui function, , with the contribution of the bonded hydrogen atom summed in, for the reaction: **13** + ⸱*i*-Pr. represents the five likely reactive carbons in **13**, carbons labelled as C2-C6, see Scheme 2. No products were isolated experimentally. The kinetically most reactive atoms as computed using the enthalpy of activation barriers, Δ, are C6 and C4⪅C5 with Δ of 47.74 kJ/mol, 54.05 kJ/mol, and 55.90 kJ/mol, respectively.

a) First choice

|  | 1 | 0.8 | | 0.6 | 0.4 | 0.2 | | 0 | -0.2 | -0.4 | | -0.6 | -0.8 | -1 | | -1.2 | -1.4 |
| --- | --- | --- | --- | --- | --- | --- | --- | --- | --- | --- | --- | --- | --- | --- | --- | --- | --- |
| -0.2 | -0.144 | -0.124 | | -0.105 | -0.085 | -0.065 | | -0.050 | -0.036 | -0.022 | | -0.008 | 0.006 | 0.020 | | 0.027 | 0.030 |
| 0.0 | -0.144 | -0.130 | | -0.115 | -0.101 | -0.087 | | -0.072 | -0.058 | -0.043 | | -0.029 | -0.014 |  | | 0.001 | 0.002 |
| 0.2 | -0.144 | -0.135 | | -0.126 | -0.117 | -0.108 | | -0.099 | -0.090 | -0.081 | | -0.072 | -0.067 | -0.073 | | -0.079 | -0.085 |
| 0.4 | -0.144 | -0.141 | | -0.137 | -0.133 | -0.130 | | -0.126 | -0.123 | -0.119 | | -0.120 | -0.133 | -0.146 | | -0.159 | -0.172 |
| 0.6 | -0.144 | -0.146 | | -0.148 | -0.150 | -0.151 | | -0.153 | -0.155 | -0.157 | | -0.178 | -0.198 | -0.219 | | -0.239 | -0.260 |
| 0.8 | -0.144 | -0.151 | | -0.159 | -0.166 | -0.173 | | -0.180 | -0.187 | -0.208 | | -0.236 | -0.264 | -0.292 | | -0.319 | -0.347 |
| 1.0 | -0.144 | -0.157 | | -0.169 | -0.182 | -0.195 | | -0.207 | -0.224 | -0.259 | | -0.294 | -0.329 | -0.364 | | -0.400 | -0.435 |
| C2 | | | C3 | | | | C4 | | | | C5 | | | | C6 | | |

b) Second choice

|  | 1 | 0.8 | | 0.6 | 0.4 | 0.2 | | 0 | -0.2 | -0.4 | | -0.6 | -0.8 | -1 | | -1.2 | -1.4 |
| --- | --- | --- | --- | --- | --- | --- | --- | --- | --- | --- | --- | --- | --- | --- | --- | --- | --- |
| -0.2 | -0.120 | -0.106 | | -0.092 | -0.078 | -0.064 | | -0.045 | -0.025 | -0.005 | | 0.014 | 0.020 | 0.023 | | 0.033 | 0.047 |
| 0.0 | -0.120 | -0.108 | | -0.096 | -0.084 | -0.072 | | -0.060 | -0.048 | -0.036 | | -0.024 | -0.012 |  | | 0.001 | 0.003 |
| 0.2 | -0.120 | -0.110 | | -0.100 | -0.090 | -0.080 | | -0.070 | -0.060 | -0.060 | | -0.061 | -0.063 | -0.061 | | -0.062 | -0.063 |
| 0.4 | -0.120 | -0.112 | | -0.104 | -0.096 | -0.088 | | -0.089 | -0.096 | -0.106 | | -0.115 | -0.116 | -0.123 | | -0.129 | -0.136 |
| 0.6 | -0.120 | -0.114 | | -0.108 | -0.101 | -0.107 | | -0.120 | -0.137 | -0.157 | | -0.159 | -0.171 | -0.184 | | -0.197 | -0.210 |
| 0.8 | -0.120 | -0.116 | | -0.112 | -0.112 | -0.131 | | -0.153 | -0.181 | -0.195 | | -0.207 | -0.226 | -0.245 | | -0.265 | -0.284 |
| 1.0 | -0.120 | -0.118 | | -0.115 | -0.131 | -0.156 | | -0.189 | -0.220 | -0.232 | | -0.257 | -0.282 | -0.307 | | -0.332 | -0.357 |
| C2 | | | C3 | | | | C4 | | | | C5 | | | | C6 | | |

**Table S28**. The predicted most and second most reactive carbon atom of the condensed R-GPRI, , for a given and using the Hirshfeld population scheme to compute the atomic charge, , and the atomic radical Fukui function, , with the contribution of the bonded hydrogen atom summed in, for the reaction: **14** + ⸱CF_3_. represents the five likely reactive carbons in **14**, carbons labelled as C2-C6, see Scheme 2. Experimentally, the major products of this reaction are located at C2 and C6 with experimental yield ratio of 5:1. The kinetically most reactive atoms as computed using the enthalpy of activation barriers, Δ, are C6≲C2⪅C5 (C6≲C5) with Δ of 25.05 kJ/mol, 27.27 kJ/mol, and 27.46 kJ/mol, respectively.

a) First choice

|  | 1 | 0.8 | | 0.6 | 0.4 | 0.2 | | 0 | -0.2 | -0.4 | | -0.6 | -0.8 | -1 | | -1.2 | -1.4 |
| --- | --- | --- | --- | --- | --- | --- | --- | --- | --- | --- | --- | --- | --- | --- | --- | --- | --- |
| -1 | -0.009 | -0.021 | | -0.043 | -0.068 | -0.093 | | -0.118 | -0.143 | -0.175 | | -0.219 | -0.263 | -0.307 | | -0.351 | -0.395 |
| -0.8 | -0.009 | -0.019 | | -0.034 | -0.054 | -0.074 | | -0.094 | -0.114 | -0.134 | | -0.170 | -0.208 | -0.245 | | -0.283 | -0.321 |
| -0.6 | -0.009 | -0.016 | | -0.024 | -0.039 | -0.054 | | -0.070 | -0.085 | -0.100 | | -0.121 | -0.153 | -0.184 | | -0.216 | -0.247 |
| -0.4 | -0.009 | -0.014 | | -0.018 | -0.025 | -0.035 | | -0.045 | -0.056 | -0.066 | | -0.076 | -0.097 | -0.123 | | -0.148 | -0.174 |
| -0.2 | -0.009 | -0.011 | | -0.013 | -0.014 | -0.016 | | -0.021 | -0.027 | -0.032 | | -0.038 | -0.043 | -0.061 | | -0.081 | -0.100 |
| 0.0 | -0.009 | -0.009 | | -0.008 | -0.007 | -0.006 | | -0.005 | -0.004 | -0.003 | | -0.002 | -0.001 |  | | -0.013 | -0.026 |
| 0.2 | -0.009 | -0.006 | | -0.003 | 0.001 | 0.004 | | 0.008 | 0.011 | 0.015 | | 0.018 | 0.021 | 0.025 | | 0.028 | 0.028 |
| C2 | | | C3 | | | | C4 | | | | C5 | | | | C6 | | |

b) Second choice

|  | 1 | 0.8 | | 0.6 | 0.4 | 0.2 | | 0 | -0.2 | -0.4 | | -0.6 | -0.8 | -1 | | -1.2 | -1.4 |
| --- | --- | --- | --- | --- | --- | --- | --- | --- | --- | --- | --- | --- | --- | --- | --- | --- | --- |
| -1 | 0.006 | -0.019 | | -0.033 | -0.044 | -0.056 | | -0.088 | -0.132 | -0.168 | | -0.193 | -0.229 | -0.268 | | -0.307 | -0.345 |
| -0.8 | 0.006 | -0.014 | | -0.028 | -0.037 | -0.046 | | -0.057 | -0.095 | -0.132 | | -0.154 | -0.181 | -0.214 | | -0.248 | -0.281 |
| -0.6 | 0.006 | -0.009 | | -0.023 | -0.029 | -0.036 | | -0.042 | -0.058 | -0.089 | | -0.115 | -0.133 | -0.161 | | -0.189 | -0.217 |
| -0.4 | 0.006 | -0.004 | | -0.014 | -0.022 | -0.026 | | -0.030 | -0.034 | -0.046 | | -0.072 | -0.087 | -0.107 | | -0.130 | -0.153 |
| -0.2 | 0.006 | 0.001 | | -0.005 | -0.010 | -0.016 | | -0.017 | -0.019 | -0.020 | | -0.023 | -0.042 | -0.054 | | -0.071 | -0.088 |
| 0.0 | 0.006 | 0.006 | | 0.005 | 0.004 | 0.004 | | 0.003 | 0.002 | 0.002 | | 0.001 | 0.001 |  | | -0.012 | -0.024 |
| 0.2 | 0.006 | 0.010 | | 0.015 | 0.019 | 0.023 | | 0.027 | 0.032 | 0.032 | | 0.031 | 0.030 | 0.029 | | 0.028 | 0.032 |
| C2 | | | C3 | | | | C4 | | | | C5 | | | | C6 | | |

**Table S29**. The predicted most and second most reactive carbon atom of the condensed R-GPRI, , for a given and using the Hirshfeld population scheme to compute the atomic charge, , and the atomic radical Fukui function, , with the contribution of the bonded hydrogen atom summed in, for the reaction: **14** + ⸱*i*-Pr. represents the five likely reactive carbons in **14**, carbons labelled as C2-C6, see Scheme 2. No products were isolated experimentally. The kinetically most reactive atoms as computed using the enthalpy of activation barriers, Δ, are C2 and C6 with Δ of 46.05 kJ/mol, and 50.20 kJ/mol, respectively.

a) First choice

|  | 1 | 0.8 | | 0.6 | 0.4 | 0.2 | | 0 | -0.2 | -0.4 | | -0.6 | -0.8 | -1 | | -1.2 | -1.4 |
| --- | --- | --- | --- | --- | --- | --- | --- | --- | --- | --- | --- | --- | --- | --- | --- | --- | --- |
| -0.2 | -0.131 | -0.112 | | -0.093 | -0.074 | -0.054 | | -0.035 | -0.016 | 0.002 | | 0.016 | 0.023 | 0.025 | | 0.026 | 0.028 |
| 0.0 | -0.131 | -0.118 | | -0.105 | -0.092 | -0.079 | | -0.066 | -0.053 | -0.039 | | -0.026 | -0.013 |  | | -0.001 | -0.002 |
| 0.2 | -0.131 | -0.124 | | -0.117 | -0.110 | -0.103 | | -0.096 | -0.089 | -0.082 | | -0.0x75 | -0.068 | -0.061 | | -0.054 | -0.057 |
| 0.4 | -0.131 | -0.131 | | -0.130 | -0.129 | -0.128 | | -0.127 | -0.126 | -0.125 | | -0.124 | -0.124 | -0.123 | | -0.122 | -0.121 |
| 0.6 | -0.131 | -0.137 | | -0.142 | -0.147 | -0.153 | | -0.158 | -0.163 | -0.168 | | -0.174 | -0.179 | -0.184 | | -0.189 | -0.195 |
| 0.8 | -0.131 | -0.143 | | -0.154 | -0.166 | -0.177 | | -0.188 | -0.200 | -0.211 | | -0.223 | -0.234 | -0.245 | | -0.257 | -0.268 |
| 1.0 | -0.131 | -0.149 | | -0.167 | -0.184 | -0.202 | | -0.219 | -0.237 | -0.254 | | -0.272 | -0.289 | -0.307 | | -0.324 | -0.342 |
| C2 | | | C3 | | | | C4 | | | | C5 | | | | C6 | | |

b) Second choice

|  | 1 | 0.8 | | 0.6 | 0.4 | 0.2 | | 0 | -0.2 | -0.4 | | -0.6 | -0.8 | -1 | | -1.2 | -1.4 |
| --- | --- | --- | --- | --- | --- | --- | --- | --- | --- | --- | --- | --- | --- | --- | --- | --- | --- |
| -0.2 | -0.120 | -0.103 | | -0.085 | -0.068 | -0.051 | | -0.033 | -0.016 | 0.004 | | 0.019 | 0.023 | 0.029 | | 0.036 | 0.043 |
| 0.0 | -0.120 | -0.108 | | -0.096 | -0.084 | -0.072 | | -0.060 | -0.048 | -0.036 | | -0.024 | -0.012 |  | | 0.001 | 0.001 |
| 0.2 | -0.120 | -0.113 | | -0.107 | -0.100 | -0.093 | | -0.087 | -0.080 | -0.073 | | -0.067 | -0.060 | -0.054 | | -0.053 | -0.047 |
| 0.4 | -0.120 | -0.119 | | -0.117 | -0.116 | -0.115 | | -0.114 | -0.112 | -0.111 | | -0.110 | -0.108 | -0.107 | | -0.106 | -0.115 |
| 0.6 | -0.120 | -0.124 | | -0.128 | -0.132 | -0.136 | | -0.140 | -0.144 | -0.148 | | -0.153 | -0.157 | -0.161 | | -0.165 | -0.173 |
| 0.8 | -0.120 | -0.129 | | -0.139 | -0.148 | -0.158 | | -0.167 | -0.176 | -0.186 | | -0.195 | -0.205 | -0.214 | | -0.224 | -0.233 |
| 1.0 | -0.120 | -0.135 | | -0.149 | -0.164 | -0.179 | | -0.194 | -0.209 | -0.223 | | -0.238 | -0.253 | -0.268 | | -0.283 | -0.297 |
| C2 | | | C3 | | | | C4 | | | | C5 | | | | C6 | | |

**Figure S5.** Linear correlation between calculated activation barriers and R-GPRI values for each reactive site in **1**. Right and left panels correspond to **⸱**CF_3_ and **⸱***i*-Pr, respectively.

**Figure S6.** Linear correlation between calculated activation barriers and R-GPRI values for each reactive site in **2**. Right and left panels correspond to **⸱**CF_3_ and **⸱***i*-Pr, respectively.

**Figure S7.** Linear correlation between calculated activation barriers and R-GPRI values for each reactive site in **3**. Right and left panels correspond to **⸱**CF_3_ and **⸱***i*-Pr, respectively.

**Figure S8.** Linear correlation between calculated activation barriers and R-GPRI values for each reactive site in **4**. Right and left panels correspond to **⸱**CF_3_ and **⸱***i*-Pr, respectively.

**Figure S9.** Linear correlation between calculated activation barriers and R-GPRI values for each reactive site in **5**. Right and left panels correspond to **⸱**CF_3_ and **⸱***i*-Pr, respectively.

**Figure S10.** Linear correlation between calculated activation barriers and R-GPRI values for each reactive site in **6**. Right and left panels correspond to **⸱**CF_3_ and **⸱***i*-Pr, respectively.

**Figure S11.** Linear correlation between calculated activation barriers and R-GPRI values for each reactive site in **7**. Right and left panels correspond to **⸱**CF_3_ and **⸱***i*-Pr, respectively.

**Figure S12.** Linear correlation between calculated activation barriers and R-GPRI values for each reactive site in **8**. Right and left panels correspond to **⸱**CF_3_ and **⸱***i*-Pr, respectively.

**Figure S13.** Linear correlation between calculated activation barriers and R-GPRI values for each reactive site in **9**. Right and left panels correspond to **⸱**CF_3_ and **⸱***i*-Pr, respectively.

**Figure S14.** Linear correlation between calculated activation barriers and R-GPRI values for each reactive site in **10**. Right and left panels correspond to **⸱**CF_3_ and **⸱***i*-Pr, respectively.

**Figure S15.** Linear correlation between calculated activation barriers and R-GPRI values for each reactive site in **11**. Right and left panels correspond to **⸱**CF_3_ and **⸱***i*-Pr, respectively.

**Figure S16.** Linear correlation between calculated activation barriers and R-GPRI values for each reactive site in **12**. Right and left panels correspond to **⸱**CF_3_ and **⸱***i*-Pr, respectively.

**Figure S17.** Linear correlation between calculated activation barriers and R-GPRI values for each reactive site in **13**. Right and left panels correspond to **⸱**CF_3_ and **⸱***i*-Pr, respectively.

**Figure S18.** Linear correlation between calculated activation barriers and R-GPRI values for each reactive site in **14**. Right and left panels correspond to **⸱**CF_3_ and **⸱***i*-Pr, respectively.
